# A new deep learning technique reveals the exclusive functional contributions of individual cancer mutations

**DOI:** 10.1016/j.jbc.2022.102177

**Published:** 2022-06-24

**Authors:** Prashant Gupta, Aashi Jindal, Gaurav Ahuja, Jayadeva, Debarka Sengupta

**Affiliations:** 1 Department of Electrical Engineering, Indian Institute of Technology - Delhi (IIT-D), Delhi, India; 2 Department of Computational Biology, Indraprastha Institute of Information Technology - Delhi (IIIT-D), Delhi, India; 3 Yardi School of Artificial Intelligence, Indian Institute of Technology - Delhi (IIT-D), Delhi, India; 4 Department of Computer Science and Engineering, Indraprastha Institute of Information Technology - Delhi (IIIT-D), Delhi, India; 5 Center for Artificial Intelligence, Indraprastha Institute of Information Technology - Delhi (IIIT-D), Delhi, India

**Keywords:** coding variants, embedding, skip-gram, survival, drivers, AP, average precision, bi-LSTM, bi-directional Long Short Term Memory, BLAC, bidirectional long short-term memory with attention & CRCS embeddings, BLCA, bladder urothelial carcinoma, cBioPortal, cBio Cancer Genomics Portal, CGI, cancer genome interpreter, CNN, Convolutional Neural Network, COSMIC, Catalogue of Somatic Mutations In Cancer, CRCS, Continuous Representation of Codon Switches, dbSNP, single nucleotide polymorphism database, ExAC, Exome Aggregation Consortium, HCC, hepatocellular carcinoma, intOGen, Integrative Onco Genomics, LUAD, lung adenocarcinoma, nsr, negative sampling rate, OncoKB, Precision Oncology Knowledge Base, SNV, single nucleotide variant, TMB, tumor mutational burden, UEC, uterine endometrioid carcinoma, ws, small window

## Abstract

Cancers are caused by genomic alterations that may be inherited, induced by environmental carcinogens, or caused due to random replication errors. Postinduction of carcinogenicity, mutations further propagate and drastically alter the cancer genomes. Although a subset of driver mutations has been identified and characterized to date, most cancer-related somatic mutations are indistinguishable from germline variants or other noncancerous somatic mutations. Thus, such overlap impedes appreciation of many deleterious but previously uncharacterized somatic mutations. The major bottleneck arises due to patient-to-patient variability in mutational profiles, making it difficult to associate specific mutations with a given disease outcome. Here, we describe a newly developed technique Continuous Representation of Codon Switches (CRCS), a deep learning-based method that allows us to generate numerical vector representations of mutations, thereby enabling numerous machine learning-based tasks. We demonstrate three major applications of CRCS; first, we show how CRCS can help detect cancer-related somatic mutations in the absence of matched normal samples, which has applications in cell-free DNA–based assessment of tumor mutation burden. Second, the proposed approach also enables identification and exploration of driver genes; our analyses implicate *DMD*, *RSK4*, *OFD1*, *WDR44*, and *AFF2* as potential cancer drivers. Finally, we used CRCS to score individual mutations in a tumor sample, which was found to be predictive of patient survival in bladder urothelial carcinoma, hepatocellular carcinoma, and lung adenocarcinoma. Taken together, we propose CRCS as a valuable computational tool for analysis of the functional significance of individual cancer mutations.

Cancer is defined as a pathological state in which the cells undergo uncontrolled cell division. There are three main causes of human cancers: (i) hereditary, which constitutes only a small fraction of cancer incidents; (ii) exposure to environmental mutagens and radiation; (iii) random errors caused during DNA replication. A recent study has indicated that about two-thirds of the cancer mutations are attributable to random errors due to defects in replication fidelity (1). Depending upon their contribution to cancer development, cancer-related somatic mutations are of two broad types—drivers and passengers. While driver mutations confer a fitness advantage to cancer cells, passengers, aka. “hitchhikers,” don't. Passenger mutations comprise about 97% of somatic mutations in cancer (2). Recent shreds of evidence highlight the indirect and damaging roles of passenger mutations (2). While a small number of driver mutations may be frequent and concentrated around driver genes, the large majority of cancer-related mutations are indistinguishable from germline variants.

Whole genome sequencing and whole exome sequencing of cancer DNA have become mainstream in cancer biology research and clinical investigations. Extensive sequencing of the cancer genomes during the last several years led to the identification and cataloging of millions of cancer-related somatic mutations that collectively allow identification of the cancer-associated mutational signatures. These mutational signatures primarily comprise substitution and frameshift mutations with one or two flanking 5′ and 3′ nitrogenous bases. These signatures are found to be differentially enriched across cancer types. The mutational signatures mainly focus on highly repetitive patterns and not much on the rare mutations which constitute the vast majority (3, 4). Further, these signatures feature the subject mutation at the center of the nucleotide string, thereby diminishing their generalizability. While all the existing approaches represent a remarkable step toward finding tractable repeating patterns across cancer genomes, these are not meant to predict cancer mutations in contrast to germline or other noncancer somatic mutations.

Accurate replication of DNA prior to cell division is crucial to impede mutagenesis. The fidelity of eukaryotic DNA replication is partially attributable to the recognition and removal of mispaired nucleotides (proofreading) by the exonuclease activity of DNA polymerases *PLOD1* and *POLE*. Church *et al.* (5) reported *POLE* mutations in the highly conserved residues, which may be strongly implicated in the impairment of the proofreading mechanism. Moreover, multiple studies linked the role of *APOBEC* cytidine deaminases in *APOBEC*-mediated mutagenesis in multiple cancer types (6, 7). Such sporadic, albeit numerous, findings collectively hint toward the exclusive nature of cancer mutations. Over the last years, the analysis of cancer genomes has primarily focused on three directions: (i) driver gene identification based on mutational recurrence; (ii) assessment of functional consequences of nonsynonymous mutations; and (iii) discovery of mutational signatures. There are two main objectives of the current study: (i) an unbiased investigation of the exclusive nature of cancer mutations as compared to germline and noncancerous mutations; (ii) demonstrating the applicability in driver gene identification and survival risk stratification in patients.

Unlike gene expressions, which are numeric, variants present the challenge of modeling categorical attributes (four nucleotides) in the context of surrounding nucleotide sequences. Latter is a more complex problem, especially since most cancer mutations are sporadic and observed in a limited number of tumor samples. A limited number of existing deep learning-based approaches enable learning from sequence data. These are used to solve diverse tasks such as unraveling regulatory motifs (8) and prioritizing functional noncoding variants, including expression quantitative trait loci in different pathological conditions (9, 10). These approaches are based on the Convolutional Neural Network (CNN) architecture. We identified two main challenges with the existing CNN-based approaches: (i) it is challenging to capture long-range dependencies by CNN that are typically expected in a DNA sequence; (ii) pooling steps in the CNN abstract the information, making it difficult for the CNNs to capture subtle differences in the sequences. To this end, we felt the urgent need for a suitable learning framework that fit the requirement of modeling functions and phenotypes associated with coding variants. A significant contribution of our work is to develop a strategy named Continuous Representation of Codon Switches (CRCS) for representing coding variants as a finite number of codon switches (total 640 in number). Further, we learned numeric embeddings (vectors of continuous values) for these codon switches, leveraging large volumes of protein-coding genetic variants observed in the population (without any known reference to any disease). Embedding of codon switches unlocks the power of the massive community-scale initiative to process and integrate nearly ∼60,000 exome sequencing profiles (11).

We constructed a novel deep learning architecture constituting bidirectional long short-term memory with attention & CRCS embeddings (BLAC) and demonstrated that a significant chunk of cancer mutations are distinguishable from noncancer mutations. We benchmarked BLAC to existing deep learning architectures and other generic methods for detecting deleterious mutations and demonstrated its power to score cancer mutations differentially. We validated our findings on independent large-scale mutational data both from cancer patients and healthy populations with no reported disorders. Our results highlight the possibility of calling somatic mutation in the absence of matched normal specimens, which has immense clinical value (12). We identified with BLAC a number of putative driver genes on the X chromosome such as *DMD*, *RSK4*, *AFF2*, *ODF1*, etc. A cumulative score was developed combining mutation level information at the patient level which showed promise in survival risk stratification in bladder urothelial carcinoma (BLCA), lung adenocarcinoma (LUAD), and hepatocellular carcinoma (HCC).

## Results

### Learning numeric representation of mutations

DNA sequences are long chains made of four types of bases, namely, adenine (A), cytosine (C), guanine (G), and thymine (T). Traditionally, machine learning–based modeling of sequence data employs one hot encoding-based presentation of each nucleotide wherein one of four possible places in a binary vector is turned 1, and the rest are set 0. Although easy to create, such a representation fails to capture the semantic relationship between two nucleotide sequences. It is possible to learn longer and more sophisticated representations, called embeddings, of nucleotides using state-of-the-art approaches (13). However, these approaches are not helpful in learning effective embeddings with such a small dictionary (consisting of four nucleotides). Alternatively, one can create embeddings of nucleotide *k*-mers (14, 15). The dictionary size of *k*-mers representation is 4^k^. Besides having large dictionary sizes, arbitrary *k*-mers do not represent biologically relevant genomic entities.

**Figure 1:**
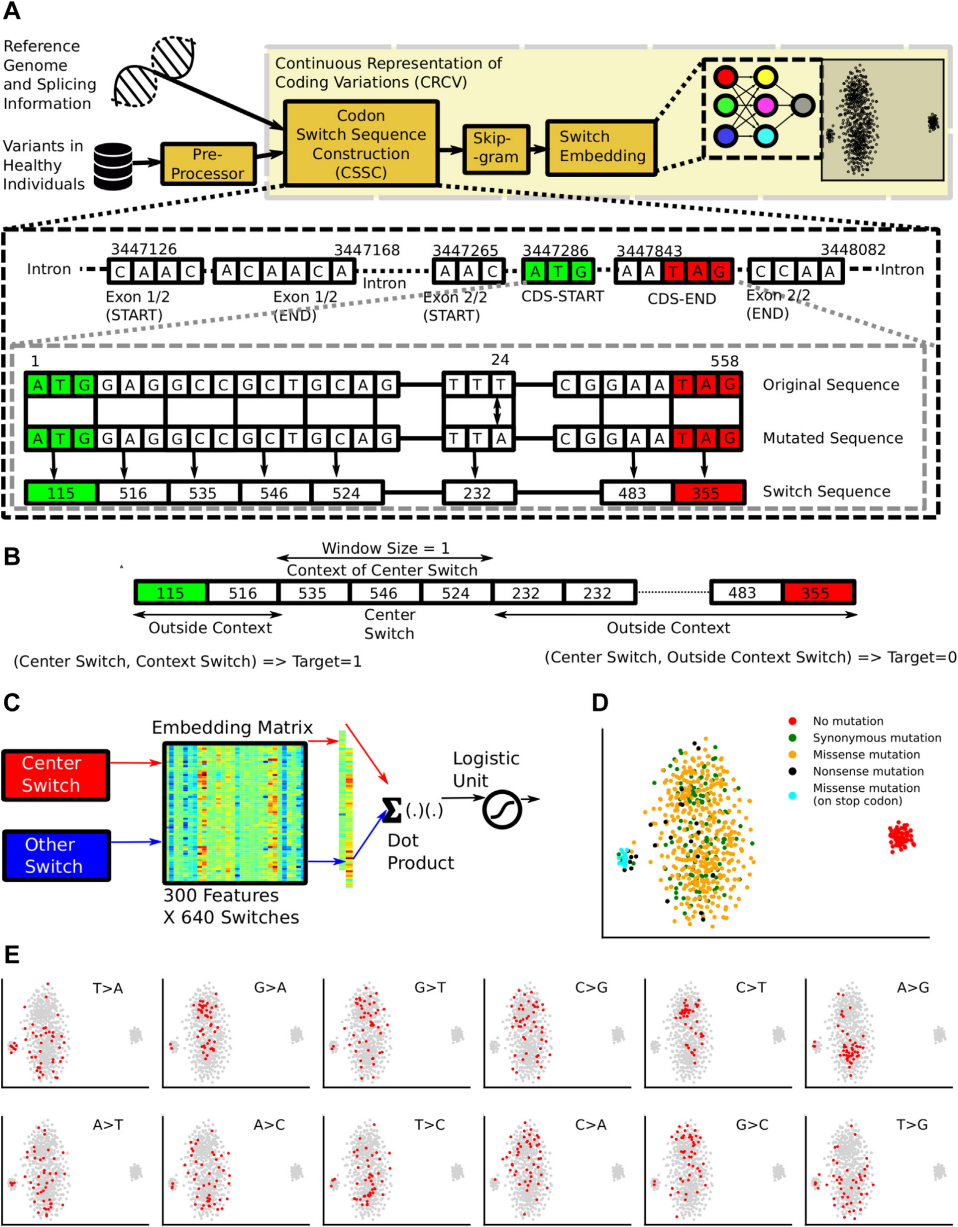
**An overview of learning Continuous Representation of Coding Switches (CRCS).***A*, the steps involved (i) selection of variants that lie in exon regions; (ii) construction of the codon switch sequence. A codon switch is defined as a directional pair of codons, constituting a reference codon (subsequence arising from the reference genome) and an alternative codon (subsequence arising from a genome of interest); (iii) creating *skip-gram* tuples; (iv) learning codon switch embedding. A toy example illustrating the process of constructing codon switch sequences is included. The number against a codon switch sequence indicates its index in the codon switch dictionary. *B*, a center codon switch is selected probabilistically. For the selected center codon switch, two types of tuples are constructed. (i) Tuples that fall within the context window of a center codon switch are marked as one; (ii) a few codon switches from outside the context are also selected, and their tuples are marked as 0. *C*, a classifier is trained to classify these tuples. Input layer weights of this network behave as codon switch embeddings. *D*, tSNE plot of learned embeddings. *E*, distribution of different codon switches on the tSNE plots. Interestingly, similar codon switches tend to cluster far from opposite codon switches (G > A and A > G, G > T and T > G, A > C and C > A, C > T and T > C). tSNE, t-distributed stochastic neighbor embedding.

This study proposes a new, biologically inspired approach to represent coding variants numerically. Coding mutations/variants can be of three types—synonymous, missense, and nonsense. This classification is based on the effect these single nucleotide variants (SNVs) have at the amino acid level. We factor this by representing coding variants as codon switches. A codon switch is defined as a directional pair of codons, constituting a reference codon (subsequence arising from the reference genome) and an alternative codon (subsequence arising from a genome of interest). Since a variant is influenced by its surrounding nucleotides, it is essential to consider the neighboring codon switches. We, therefore, constructed codon switch sequences containing the codon switch of interest for modeling purposes. For this study, we could only consider single base substitution since other categories such as frameshift and complex alterations (including double base substitutions) are sparsely present in the data (constituting less than 4% of the entire repertoire of cancer-related alterations) (Fig. S1). Figure 1*A* depicts the details of this construction process. Effectively, a codon switch does not necessarily represent an alteration, it may also represent an unaltered amino acid (*e.g.*, ATA→ATA). A dictionary of codon switches constructed in this way contains a total of 640 codon switches (Codon switch sequences). Notably, our entire study focuses on coding sequences only.

In this proof of concept study, we applied our approach on mutations in chromosome X. We only used mutations from healthy individuals to learn the embeddings. Embeddings are finite-sized numerical vectors. In order to construct mutational embeddings, the codon switch sequences were subjected to *Skip-gram* with *negative sampling* (13). *Skip-gram* is a popular shallow neural network architecture that is used in the domain of natural language processing to obtain a numeric representation of words that preserves semantic similarity between word pairs that appear in the same context across discourses. We modified the *skip-gram* network and posed the learning task as a classification problem. To train the network, we generated tokens based on the neighborhood of a codon switch from a codon switch sequence (Fig. 1, *B* and *C*). A total of 68,836 unique coding substitutions from healthy individuals (Exome Aggregation Consortium, ExAC (11)) were used for learning the numeric representation of coding codon switches. Further details on the training of codon switch sequences can be found in Continuous embedding of codon switches.

The semantic representation obtained by training the neural network was captured in 300-sized numeric vectors, representing each of the 640 codon switches. As vectors of similar words correlate strongly, codon switches that share similar nucleotide contexts orient themselves analogously in the associated vector space. Figure 1*D* shows the t-distributed stochastic neighbor embedding projections of the learned embeddings. Interestingly, codon switches without any substitution (identical codons) tend to form a separate cluster. Codon switches with mutations in the STOP codons form a separate cluster. Missense and nonsense mutations are divided into two overlapping clusters. A closer inspection of these two overlapping clusters reveals that codon switches in these clusters are localized in a complementary fashion. This shows the reversible functional impact of mirroring codon switches. Note that codon switches with G > A mutation and A > G mutation are located in different clusters. Codon switches with other complementary mutations also display similar trends (Fig. 1*E*). Fig. S2 shows UMAP projections of the learned embeddings. Notably, we could not spot any meaningful pattern among codon switches representing a change in amino acid side chain properties such as polarity and acidity (Fig. S3).

### CRCS exposes the inherent diversity of chromosomes

**Figure 2:**
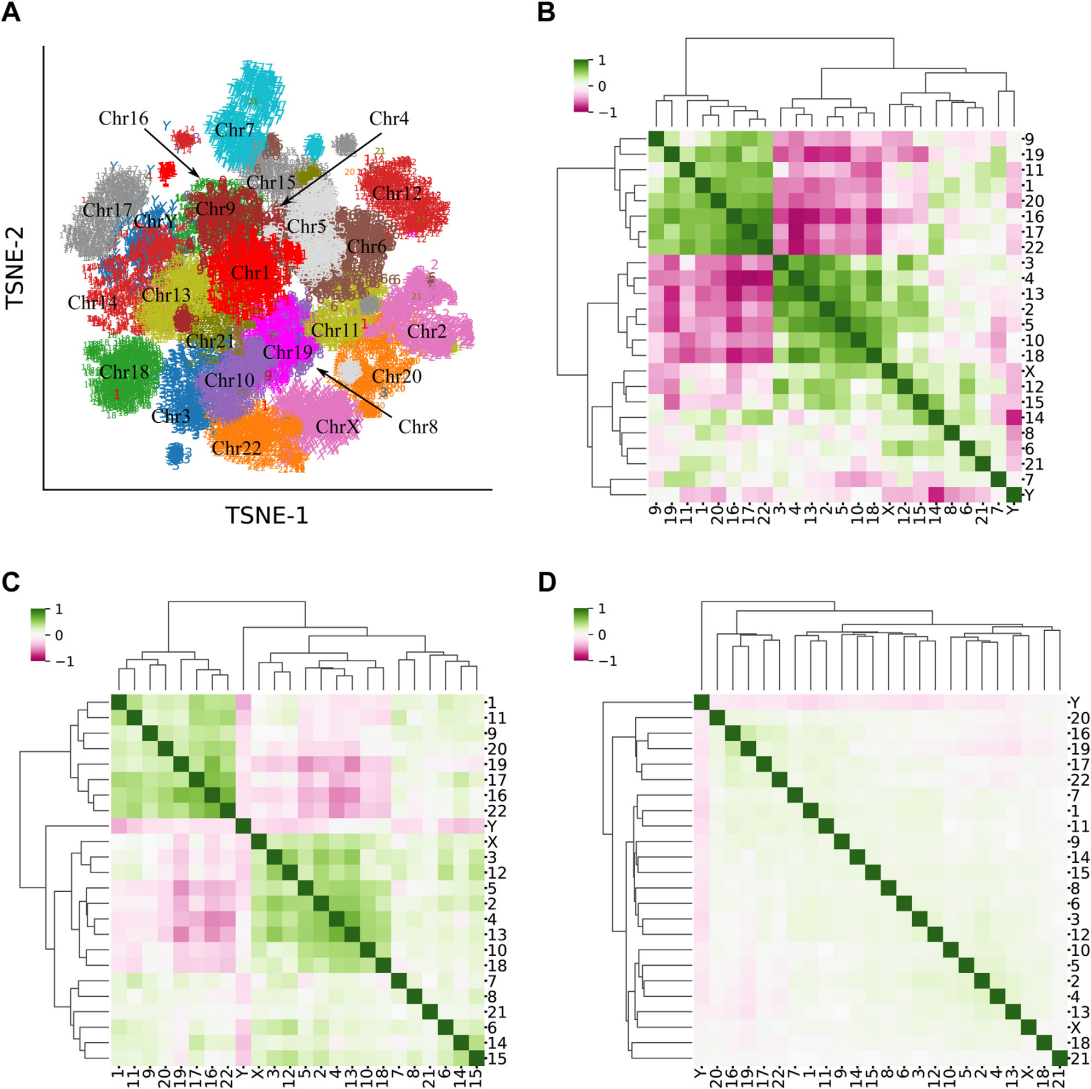
**CRCS embeddings reveal the exclusive nature of chromosomes.***A*, tSNE projections of the embeddings learned independently for all the chromosomes. The embeddings are clearly segregated, indicating heterogeneity in nucleotide sequence patterns. *B*, Spearman correlation of unigram frequencies across chromosomes. Chromosomes are found to give rise to some tight clusters. *C*, Spearman correlation of bigram frequencies in chromosomes. *D*, correlation of trigram frequencies across chromosomes. Chromosomes described as trigrams. Chromosomal similarities fade away with increase in the sequence length. CRCS, Continuous Representation of Codon Switches; tSNE, t-distributed stochastic neighbor embedding.

To analyze other chromosomes, we generated CRCSes for all the remaining chromosomes. A t-distributed stochastic neighbor embedding visualization of the chromosome-specific CRCSes highlights heterogeneity manifested by chromosomal nucleotide sequence patterns (Fig. 2*A*). To further investigate, we generated the unigrams (individual amino acids), bigrams (strictly ordered consecutive amino acid pairs), and trigrams (strictly ordered consecutive amino acid triplets) of amino acids from the sequences and analyzed chromosomal frequencies. Fig. 2*B* (Supplementary File 2) shows the similarity of chromosomes in terms of the frequency of individual amino acids. Similar figures are also generated for bigrams and trigrams (Fig. 2, *C* and *D*). Clear biases are observed among different chromosomal groups at unigram and bigram levels, suggesting amino acid composition differences. At the trigram level, such chromosomal groups start fading away. This analysis suggests that independent learning of embeddings may be necessary for other chromosomes.

Different chromosomes harbor different sets of genes that are often functionally connected to reduce cell regulatory redundancies. Examples are *HOX* and Odorant Receptor families. *HOX* genes are colocalized in chromosomes in many species, such as *Drosophila*. In humans, 39 *HOX* genes are present as clusters across four chromosomes (16). Similarly, a significant fraction of human Odorant Receptors is clustered in Chromosome 11 (17). This could be a strong reason for sequence bias across chromosomes. Chromosomal sequence biases can also be explained by local gene duplication (18). Taken together, our analysis unravels inherent differences in nucleotide sequence patterns across human chromosomes, which demands further investigation. It is also apparent that machine learning models should be created in a chromosome-specific manner to enable various genotype-phenotype association studies.

### Classifying cancerous and noncancerous mutations

Identifying cancer mutations is vital in various clinical settings, albeit challenging. The most common use case is detecting somatic mutations from tumor specimens in the absence of matched normal tissue (12). This causes the under-utilization of clinical sequencing outputs. On a separate note, tumor mutational burden (TMB) is estimated by counting cancer-related somatic mutations from cancer specimens. A robust pipeline for cancer mutation detection includes the subtraction of germline variants obtained from matched normal samples. TMB estimation has been proven to be an efficient way to monitor cancer treatment (19). Due to the challenges involved in obtaining tissue biopsies, it is crucial to assess TMB using cell-free DNA from blood, which may include circulating tumor DNA. In the absence of matched normal samples, the germline variant databases are used for *in-silico* filtering. These methods are suboptimal and can benefit significantly from the normal-free detection of cancer mutations. We investigated if a classifier can be trained to classify cancerous and noncancerous mutations.

We used CRCSes to classify codon switch sequences into two categories, namely codon switch sequences harboring cancerous mutations or noncancerous mutations. Due to the significant computational overhead, we focused on the sex chromosomes for downstream analysis. We note that ∼70 protein-coding genes harbored by chromosome Y offer inadequate levels of genetic diversity, thereby trivializing deep learning-based interventions. On the other hand, we obtained about 107,000 high-quality variants across ∼800 genes from the ExAC browser for chromosome X. We considered splicing events when populating codon switch sequences for training a custom neural-network architecture for classification. Notably, we generated embeddings for all 640 codon switches independently for each chromosome, and we found substantial heterogeneity, which the chromosomal amino acid composition biases can explain. As such, genome-wide applicability of CRCS warrants independent model building for each specific chromosome (Fig. 2).

**Figure 3:**
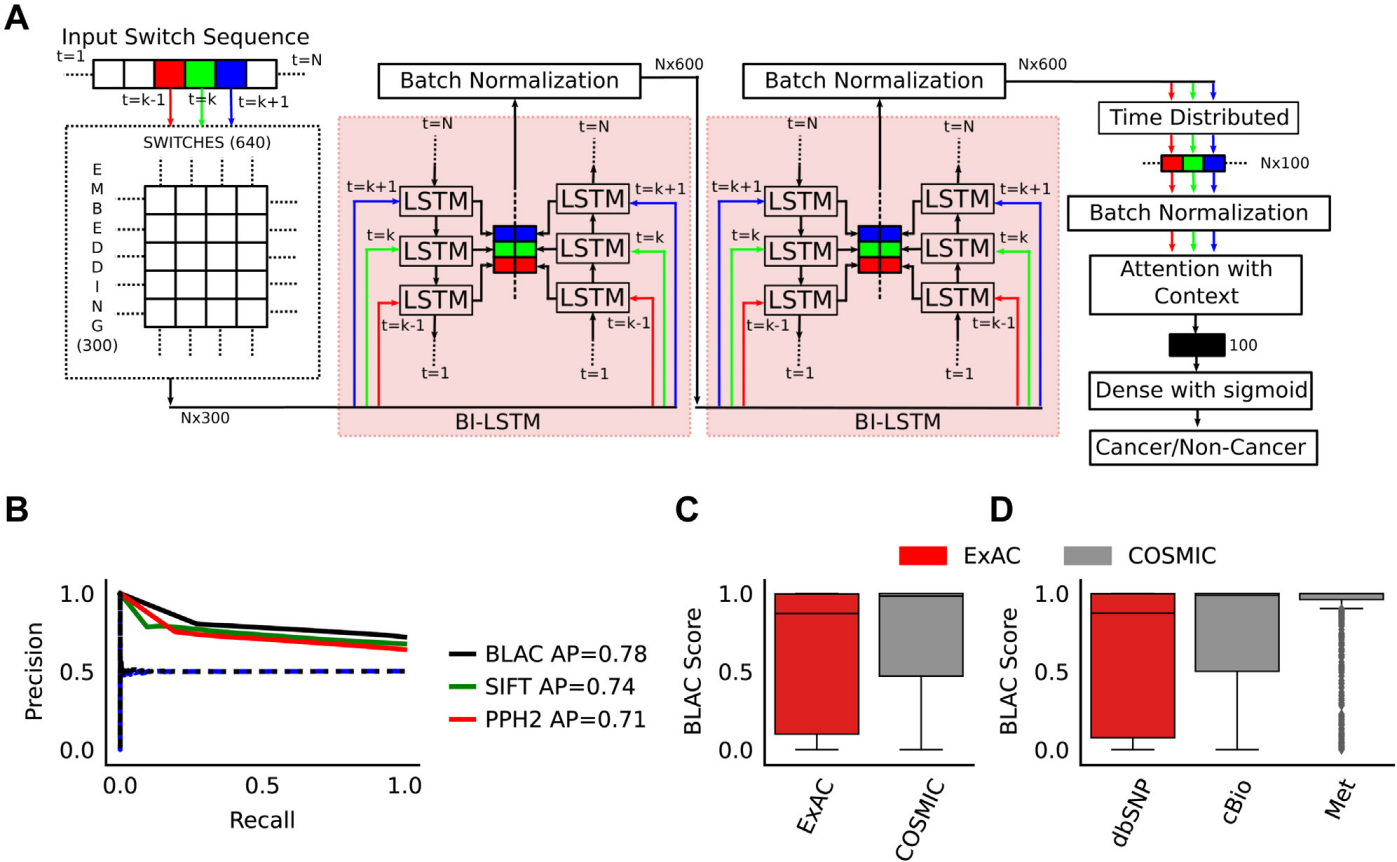
**Classification of cancerous and noncancerous variants.***A*, deep learning architecture, used for CRCS-based classification of ExAC/COSMIC variants. *B*, Precision-Recall (PR) curve for the BLAC after 200 epochs. The *red* and *green curves* indicate the performance of SIFT (20) and Polyphen2 (21), respectively. Validation performances were measured on fake alteration classes, constructed by randomly splitting cancer/noncancer alterations into two equal-size groups. The *black dashed line* represents the performance of the fake test set created from COSMIC data. Similarly, the *blue dashed line* is for ExAC data. Both PR curves thus obtained, as expected, collapsed on the 0.5 precision line. *C*, boxplots depict the distribution of prediction scores (probability of being a cancer alteration), assigned to the ExAC and COSMIC alterations, in the validation set (across all folds). *D*, similar trends are observed for nonpathogenic dbSNP alterations and mutations found in cancer patients from Met (23) and cBioPortal (62, 63). Scores on these datasets were predicted using the model trained on the full dataset. BLAC, bidirectional long short-term memory with attention & CRCS embeddings; COSMIC, Catalogue of Somatic Mutations In Cancer; CRCS, Continuous Representation of Codon Switches; ExAC, Exome Aggregation Consortium.

For predicting noncancerous/cancerous mutations, we trained our custom neural network architecture, BLAC, using 34,981/66,165 unique X-chromosome specific substitutions from ExAC/Catalogue of Somatic Mutations In Cancer (COSMIC) databases spanning 332 protein-coding genes (Fig. 3*A*). Four-fold cross validation was used to evaluate the performance of BLAC-based detection of cancer mutations.

We obtained an average precision (AP) (*i.e.*, the area under the precision-recall curve) of 0.78 on the four validation sets, indicating predictability of the mutational subtypes (Fig. 3*B*). We posed a similar classification problem by randomly splitting the SNV pool obtained from the ExAC browser as a control. As expected, we obtained an AP of 0.5. With COSMIC alterations, our finding was similar (Fig. 3*B*). This strongly supports the conclusion that cancer-related somatic mutations are surrounded by differential nucleotide contexts compared to noncancerous variants. We compared the performance of the trained model with SIFT (20) and Polyphen2 (21). These methods are widely used to predict the deleterious nature of mutations, using sequence homology and amino acids' physical properties. SIFT and Polyphen2 yielded lower values of AP (0.74 and 0.71, respectively), indicating the superiority of our sequence-based approach. Notably, BLAC predictions are based on unseen genes (due to our implementation of cross validation), whereas SIFT and Polyphen2 use models trained on the entire genome. Under the current experimental setting, SIFT and Polyphen2 enjoy a significant relaxation in terms of the stringency of cross validation.

The median distributions of scores on ExAC and COSMIC datasets (Fig. 3*C*), although significantly different, are on the higher side of the spectrum, which has a high false-positive rate at a threshold of 0.5. Thus, there is still a large gray area in the probability spectrum where cancer-related mutations are indistinguishable from noncancerous ones. Many cancer-related mutations received poor probability scores, leading us to the postulate that only a fraction of cancer-related mutations occur amid exclusive nucleotide contexts. Choosing a threshold value of 0.9, although it reduces the model's sensitivity, returns the mutations that have high chances of being cancerous. Table S1 (and Fig. S4) shows the value of specificity, sensitivity, and F1-score at the threshold of 0.9. It is evident from the table that Polyphen2 has higher specificity while BLAC scores have higher sensitivity and F1-score.

To independently validate our findings, we evaluated our model trained on ExAC/COSMIC on three different datasets. We took neutral SNVs from the single nucleotide polymorphism database (dbSNP) after removing entries tagged as pathogenic (22). We considered somatic SNVs from the Met study for a matching cancer alteration pool, a recently published pan-cancer study of solid metastatic tumors (23). Met sequenced and analyzed 2520 Dutch population tumor samples. We followed the filtering criteria discussed in Pruning of the coding variants. After removing sequences overlapping with the training set, 289,418 noncancer- and 1151 cancer-related mutations were left. We used cBio Cancer Genomics Portal (cBioPortal) as the second source of cancer mutation data for validation. All 287 studies were downloaded, and chromosome X mutations were chosen. After applying our filters as discussed above, 147,049 mutations were left from the original 246,201. As expected, cancer mutations were assigned relatively higher BLAC scores (Mann-Whitney U-test *p*-value < 0.01), thereby underscoring the robustness and crossdemographic reproducibility of our predictions (Fig. 3*D*).

While interoperability between chromosomes appears intuitive, it might not be optimal for the discussed classification task. As discussed earlier in the section, the embeddings of codon switches are well segregated, indicating apparent heterogeneity (Fig. 2). To further reinforce this, we predicted the BLAC scores on chromosome 22 using the embeddings and classification model trained on chromosome X. As expected, the results on these values were inferior than the chromosome-specific model (Fig. S5).

We also compared the proposed neural network with an alternate embedding approach, that is, dna2vec (14) and found its performance to be inferior to that of the CRCS-based method (Fig. S6, *A* and *B*). Further, we also compared our network with three different architectures, namely, DanQ (8), DeepSea (9), and HeartENN (10). These methods also failed to supersede the performance of the CRCS-based approach (Fig. S6, *C* and *D*). A detailed discussion of this validation can be found in Supplementary Note S1.

### BLAC score assists in driver gene exploration

**Figure 4:**
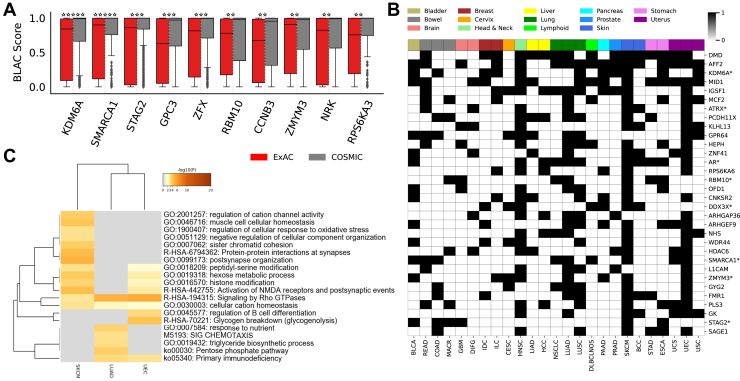
**Driver gene analysis and exploration.***A*, boxplots show the distribution of prediction scores assigned to ExAC and COSMIC alterations for the known driver genes from the validation set (across all folds). In the figure, five *stars* represent a *p*-value less than 5*e*^−15^. Values in the range [5*e*^−15^, 5*e*^−12^) are represented by four *stars*. Similarly, values in the range of [5*e*^−12^, 5*e*^−9^), [5*e*^−9^, 5*e*^−6^), and [5*e*^−6^, 5*e*^−2^) are represented by 3, 2, and 1 *stars*, respectively. *B*, heatmap shows the genes (in *black*) that have been marked significant most frequently, across cancer types. For a given cancer type in cBioPortal, a gene was marked significant if the BLAC scores of the reported mutations were significantly elevated as compared to dbSNP variants. The colors in the *top row* show the organ of cancer. Gene marked with ∗ are known driver genes. *C*, heatmap depicting the cluster-wise enrichment of the prominent biological functions in the indicated cancer types. Of note, the selected cancer types harbored a number of mutational genes identified using the CRCS-based approach. Cancer types that displayed significantly divergent risk groups include skin cutaneous melanoma (SKCM), lung adenocarcinoma (LUAD), and undifferentiated endometrial carcinoma (UEC). The scale bar represents the negatively log-transformed (base 10) *p*-values. BLAC, bidirectional long short-term memory with attention & CRCS embeddings; COSMIC, Catalogue of Somatic Mutations In Cancer; CRCS, Continuous Representation of Codon Switches; dbSNP, single nucleotide polymorphism database; ExAC, Exome Aggregation Consortium.

Driver genes play a pivotal role in the diagnosis and clinical management of cancers. We asked if our model differentiates between driver gene-specific noncancerous and cancerous mutations. By merging multiple driver gene databases (Precision Oncology Knowledge Base, (OncoKB) (24), Integrative Onco Genomics, (intOGen) (25), and cancer genome interpreter (CGI) (26)), we obtained 55 potential driver genes on chromosome X, of which 33 were left after filtering. For these 33 driver genes, ∼148 and ∼680 coding variants were retrieved, on average, from ExAC and COSMIC, respectively. On feeding these variants to our CRCS pipeline, we observed significant differences in the distribution of prediction scores. Figure 4*A* presents the top ten genes (*KDM6A*, *SMARCA1*, *STAG2*, *GPC3*, *ZFX*, *RBM10*, *CCNB3*, *ZMYM3*, *NRK*, and *RPS6KA3*) based on *p*-values. The distribution of scores for the remaining 23 genes is presented in Fig. S7.

We asked if the strength of differential elevation of BLAC scores between cancerous and noncancerous mutations is more pronounced in the case of cancer drivers. For this, we computed the statistical significance of BLAC score differences associated with all genes (on Chromosome X) by leveraging cBioPortal (for cancer mutations) and dbSNP (noncancerous) variant calls (Comparing cBioPortal predictions with dbSNP predictions, Supplementary File 3). We found adjusted *p*-values (we considered −log10 transformation of the adjusted *p*-values in this case) associated with the known driver genes to be of higher significance than the entire population of X chromosome-specific genes (one-sided Kolmogorov-Smirnov test *p*-value < 0.05). This indicates that one could use the differential elevation of BLAC scores across cancerous and noncancerous mutations for a given gene as a yardstick for its driver potential. Figure 4*B* reports 32 genes that show significant, cancer-specific BLAC score elevation across five or more cancers. Out of 32 genes, 24 were not reported in either of the three databases: OncoKB (24), intOGen (25), and CGI (26). Among the genes not cataloged in these three databases, *DMD* is an important candidate. Mutation of the *DMD* gene causes muscular disorders. However, increasing shreds of evidence implicates *DMD* in developing all major cancer types (27). *RPS6KA6* (aka *RSK4*) has recently been found to play a pivotal role in promoting cancer-stem-cell properties and radioresistance in esophageal squamous cell carcinoma (28). BLAC score–based analyses indicated its potential involvement in pancreas, liver, head-and-neck, and breast cancers. Another intriguing candidate is *OFD1*, a protein involved in ciliogenesis (29). The primary cilium is a thin and long organelle protruding in almost all mammal cell types and is involved in perceiving external stimuli, such as light, odorants, and fluids. The primary cilium also coordinates signaling pathways that convert extracellular cues into cellular responses with the help of receptors and signaling molecules. *OFD1* mutations have been found implicated in Wnt hyper-responsiveness (30). *WDR44*, another enlisted gene, is involved in ciliogenesis (31). Its role in cancer is still elusive. Supplementary File 4 cites reports highlighting the roles of the 32 genes with information on their potential as cancer drivers, where applicable. Genes such as *AFF2*, *MID1*, *PCDH11X*, *MCF2*, *NHS*, and *GYG2* are not reported to have a role in cancer pathogenesis and could be interesting for future validation. Notably, *AFF2* has recently been predicted to have driver roles (32).

We asked if genes that show differential BLAC scores across cancerous and noncancerous mutations in specific cancer types are functionally interconnected. For this, we used gene ontology analysis by Metascape (33). For the top three cancer types, that is, skin cutaneous melanoma, LUAD, uterine endometrioid carcinoma (UEC), harboring the maximum number of genes (≥100) identified by the method discussed in Comparing cBioPortal predictions with dbSNP predictions (Supplementary File 5). Metascape-based functional enrichment analysis revealed the contribution of identified genes to be largely cancer-specific (Fig. 4*C*). For example, the pentose-phosphate pathway (34, 35) and the triglyceride biosynthesis process (36) are highly enriched in LUAD. Similarly, the glycogenolysis pathway is enriched in UEC (37). We relaxed the number of gene cutoff to ≥40 and obtained seven cancer types (Supplementary File 5), namely skin cutaneous melanoma, lung squamous cell carcinoma, UEC, LUAD, head and neck squamous cell carcinoma, BLCA, non–small cell lung cancer, classified based on the number of genes they possess (Fig. S8). Similar to our earlier analysis, we observed cancer-specific pathway enrichments, suggesting functional interconnections between identified genes (Fig. S9). For instance, in the case of BLCA, we observed a specific enrichment for the carbon metabolism pathway (38, 39, 40). The results suggest that genes that attract more deleterious/driver-like mutations in specific cancers selectively alter different pathways. For example, modifications in the histone pathways are well characterized in multiple cancer types (41, 42).

### BLAC scores enable survival risk stratification in different cancer types

Characterization of tumor specimens using next-generation sequencing is becoming increasingly common in targeted treatment selection. These processes offer large numbers of alterations per patient. A significant technical difficulty in detecting all somatic mutations from a tissue sample is that it requires the availability of matched normal tissue samples. In practice, paired collection of cancer and normal tissue samples is quite challenging. Even if all somatic mutations are detected, it is hard to draw any conclusion unless these are characterized. As such, presently, only a small fraction of these, which are well characterized, is finally taken into account to devise therapeutic strategies (12).

Since the advent of massively parallel sequencing platforms, numerous sophisticated methods have been developed for the stratification of patients with differential prognoses. Most of these methods map missense mutations to genes, thereby losing their individualities. For example, Hofree *et al.* (43), in a seminal paper, mapped somatic mutations to gene networks to cluster tumors (genome sequences) after network smoothing using random walk with restart. Clusters of patients thus obtained indicated significantly differential survival patterns. Milanese *et al.* (44) leveraged putative functional mutations to predict recurrence in breast cancer. Their approach is also based on mapping mutations to genes. We hypothesized that CRCS could be used for risk stratification using mutation-level information only.

**Figure 5:**
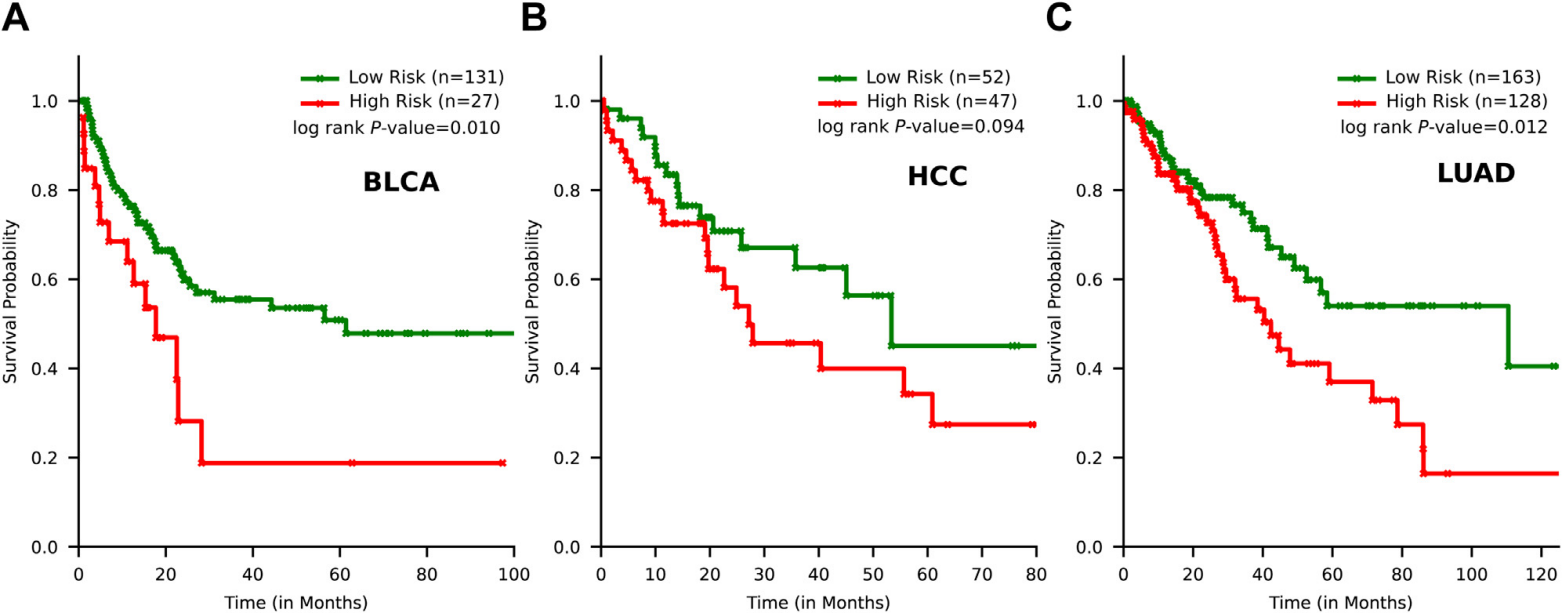
**Survival risk stratification based on cumulative BLAC scores.***A*, patients with lower average BLAC scores in bladder urothelial carcinoma (BLCA), a subtype of bladder cancer, have better survival. *B* and *C*, similar trends are also visible in hepatocellular carcinoma (HCC), a subtype of liver cancer, and lung adenocarcinoma (LUAD), a subtype of lung cancer. BLAC, bidirectional long short-term memory with attention & CRCS embeddings.

Our construction of cancer and noncancer mutation classification problem unavoidably discounts the fact that some cancer-related somatic mutations could indeed be randomly located and hard to differentiate from other germline mutations. This could be the primary reason for the overlapping BLAC scores associated with the two categories. We, therefore, inferred that mutations with extremely high BLAC scores might indicate a higher degree of contribution to the cancer hallmarks. We devised a cumulative BLAC score based on individual BLAC scores associated with X chromosome–specific mutations (since our demonstration of CRCS is limited to X chromosomes only) such that mutations with higher BLAC scores are given relatively higher weightage (using an exponential function). We performed this task on cBioPortal mutations that are not used in training our prediction model. Out of eight cancer types that qualified our data filtering criteria, three showed significant (log-rank *p*-value < 0.1) survival risk stratification based on this score (Fig. 5). The three cancer types are BLCA, HCC, and LUAD. In each case, patients with higher cumulative BLAC scores were mapped to the high-risk group. Significant aberration of the X chromosome has already been reported in HCC (https://pesquisa.bvsalud.org/portal/resource/pt/wpr-520359, (45)). In BLCA, similar reports exist highlighting the association of *KDM6A* hypermutation with antitumor immune response efficacy (46, 47, 48, 49). Notably, BLAC scores of cancer-associated somatic mutations in *KDM6A* show higher elevation than germline or other noncancerous somatic mutations (Fig. 4*A*).

## Discussion

The majority of cancer mutations are thus far understood as hitchhikers. No notable computational work convincingly indicates the contextual difference between cancerous and noncancerous mutations. This problem has great use in clinics since often matched normal sequences are not available for confident somatic mutation calling. A good example is detecting mutations in cell-free DNA for diagnostic purposes or measuring the mutational burden post-chemotherapy or immunotherapy (50). Furthermore, current computational approaches for risk stratification of cancer patients under-utilize information captured at the mutation level. These methods typically map missense mutations to genes for further downstream prediction tasks. The current work reports a strategy to address this by learning numeric vector-based representations of mutations (*i.e.*, embeddings) that are more amenable to machine learning tasks.

It has been observed that cancer mutations occur at evolutionarily conserved sites (51) and active sites of proteins (52). Further, mutational signatures (*i.e.*, specific alterations surrounded by specific flanking nucleotides) have been identified and correlated with different mutagenesis processes such as smoking and UV exposure (53). However, no holistic approach is visible to discriminate between cancerous and noncancerous variants. In fact, it is still elusive whether cancer mutations are exclusive in nature. The current work answers this in the affirmative.

Currently, there is no mainstream strategy that embeds the individual alterations while preserving their similarity in terms of the nucleotide sequence context. One visible effort in this direction is mut2vec (54), which provides mutational embeddings at the gene level, leveraging text mining, and protein–protein interaction network. This learning approach does not mimic any underlying biological process. For the first time, our approach enables learning semantic representation of mutations from large volumes of uniformly processed exome sequencing data.

Our CRCS and bi-directional Long Short Term Memory (bi-LSTM)–based custom deep learning architecture (BLAC) can discriminate between cancerous and noncancerous mutations with a limited number of labeled samples from each category. This is due to the powerful representation learning accrued by the CRCSes by ingesting a large pool of SNVs from tens of thousands of exome sequencing data. The prediction is not black and white as the BLAC score spectrum associated with cancerous and noncancerous mutations overlap substantially. However, we see an apparent elevation in the distribution of CRCS scores for cancer mutations. This provides evidence for the exclusive nature of cancer mutations and the fact that cancer mutations, by and large, differ from germline or other somatic mutations in terms of nucleotide context.

Most of the existing deep learning architectures proposed for solving various tasks using sequence data use CNN as the building block. There are a few fundamental problems in using a CNN layer to solve the problem as put forth in the article. (i) In the CRCS representation, there is only one codon switch in the sequence containing actual mutational information. Utilizing CNN followed by a max-pooling on this representation may drop the variability introduced by a single codon switch. Thus, it becomes challenging for the model to differentiate among mutations on an mRNA. (ii) A CNN layer is not able to handle variable-length sequences. The maximum length of the mRNA sequence needs to be fixed at the input, and then other variable-length sequences need to be padded with zeros to be fed into the network. This restriction on the input size limits the generalizability of the model.

In contrast to CNNs, LSTMs are specifically designed to capture long-range dependencies. Single position difference in the codon switch sequence can alter the state of the LSTM unit, ultimately changing the prediction of the network. Bi-LSTMs, a variant of LSTM, look at both ends of the sequence together, which further improves the prediction of the network. In addition, the recurrent nature of LSTMs makes them suitable for variable-length sequences. Apart from these, in other proposed networks, concatenation of one-hot encoded nucleotides was used to create the numerical representation of the DNA sequence. Such representations are not able to capture any semantic information. We realized that these problems prohibit unlocking the information content present in variable-length flanking regions surrounding the coding variant of interest.

The current work reports results only on chromosome X. This study can also be extended to the other chromosomes. Given that CRCS embeddings vary across chromosomes (Fig. 2), in future work, we plan to train separate models for all the chromosomes, for genome-scale analysis of mutations. Further, we need to devise strategies to accommodate minority variants such as indels and complex alterations.

In a nutshell, our findings suggest that cancer-specific SNVs, including passenger mutations, occur with differential nucleotide contexts compared to coding variants observed in healthy populations. One significant advantage of CRCS is that it is not reliant on any clinical or pathological parameters. We predict that the proposed approach could be adopted in attempting a broad range of questions concerning genotype-phenotype interlinking.

Mendelian diseases such as sickle cell anemia or cystic fibrosis can be linked with variants in single genes. On the contrary, complex diseases such as myocardial infarction or schizophrenia result from combinatorial effects of multiple genetic variants (55). Genome-wide association studies are popular in linking genotypes with diverse conditions or traits. As an outcome of these studies, over 10,000 variants (common in most cases) have been identified and associated with various diseases during the last 15 years (56). In genome-wide association studies, stringent false discovery rate control comes at the expense of true positive discovery or statistical power (57). A substantial number of variants are ignored due to lower frequencies. Numerical vector-based representation using CRCS may be able to rescue these otherwise ignored variants. According to the World Health Organization definition, rare diseases affect fewer than one in 200,000 people in the USA, and there are as many as 7000 such diseases (https://my.clevelandclinic.org/health/diseases/21751-genetic-disorders). Amyloidosis, Adrenoleukodystrophy (connected to X chromosome), and mitochondrial diseases fall under this category. Due to sample size issues and low minor allele frequencies, many rare diseases cannot easily be linked with variants. CRCS approach can be fruitful in such cases. This approach can also be used to train models that can identify previously uncharacterized deleterious mutations that can lead to structural changes to protein and corroborate computational structural biology approaches. Alzheimer's and Parkinson’s are examples of some neurodegenerative diseases that can be linked with causal variants (58). The proposed method can also be extended to understand the role of splice site point mutations and their implication in various diseases such as congenital cataracts and Becker muscular dystrophy (59).

## Experimental procedures

### Description of datasets

We collected high-quality coding SNVs representing the general population from the ExAC browser (11) (https://console.cloud.google.com/storage/browser/gnomad-public/legacy/exacv1_downloads/release1). The same data can also be downloaded from the gnomAD website (60) (https://gnomad.broadinstitute.org/downloads). An equivalent set of neutral SNVs was downloaded from the dbSNP after removing genomic alterations that are tagged *pathogenic* (22) (ftp://ftp.ncbi.nih.gov/snp/latest_release/VCF). Cancer-associated coding variants were downloaded from the COSMIC (61) (v89) and cBio Cancer Genomics Portal (cBioPortal) (62, 63).

A list of known driver genes for chromosome X was constructed by combining information from three sources - OncoKB (24), (intOGen) (25), and CGI (26). OncoKB has 44 driver genes, out of which 7 (13) are annotated as oncogenes (tumor suppressors). Among the remaining genes, 23 are not annotated. *MED12* is annotated as both an oncogene and a tumor suppressor. IntOGen reports 36 driver genes, out of which OncoKB also reports 25. Amongst these 25 genes, 3 (12) are annotated as oncogenes (tumor suppressors) by OncoKB, while the remaining are not annotated. CGI reports seven driver genes, out of which 4 (1) genes are annotated as oncogenes (tumor suppressors). One driver gene is unannotated. OncoKB also reports all genes reported by CGI. *MED12* is reported by both IntOGen and CGI Supplementary File 6.

Reference genome (hg19/GRCh37) was downloaded from the UCSC genome browser (64). The list of mRNA and their coordinates were obtained from kgXref and the knownGene tables from the UCSC table browser (65).

### Pruning of the coding variants

knownGene and kgXref tables were combined, and only protein-coding mRNAs were selected. VCF files from ExAC and COSMIC were scanned, and genomic alterations (indels plus SNVs) on the protein-coding region of the genome were analyzed further. Of 4,537,166 (4,664,549) alterations collected from ExAC (COSMIC), 107,591 (197,085) alterations were from the X-chromosome. These alterations were mapped to all possible splice variants of the mRNAs, which, for chromosome X, inflated the alteration counts to 289,813 (624,918) from ExAC (COSMIC). Since the frequency of insertions, deletions, and complex mutations have a very small contribution to the datasets (Fig. S1, Supplementary Note S2, Supplementary File 7, Table S2), we restricted the scope of our analysis to SNVs alone. To this end, 285,102 (590,171) SNVs, considering all possible splice variants harboring the SNVs, were retained from ExAC (COSMIC). Removal of duplicate SNVs caused a reduction of 21,495/308,198 in these counts corresponding to ExAC/COSMIC. After removing duplicates, 40% of variants from ExAC were kept aside to learn embeddings. The remaining counts of variants from ExAC and COSMIC were 149,566 and 281,973, respectively. For ExAC/COSMIC, the count of synonymous, missense, and nonsense variants from chromosome X were 54,541/62,102, 93,647/202,981, and 1313/16,703, respectively. Since synonymous variants are expected to have minimal effect on cellular fitness (66), they were removed from further processing. At this stage, the preprocessed data contained 94,960/219,684 variants from ExAC/COSMIC dataset. SNVs specific to dbSNP (22), Met (23), and cBioPortal (62, 63) were also preprocessed in a similar manner. In dbSNP, variants marked as pathogenic and likely-pathogenic were removed. After preprocessing, the total X chromosome–specific SNVs from dbSNP were 530,405. Across 287 studies present in cBioPortal, mutations reported on the X chromosome were filtered. After preprocessing, the mutation count was 374,138 and 2611 for cBioPortal and Met data, respectively.

### Codon switch sequences

In this article, we present a novel representation for mutations, *viz.* as codon switches. A codon switch dictionary was created by altering one nucleotide in a codon at a time. This results in 640 codon switches.† Here, we justify the count of the total number of codon switches. A codon is made of three nucleotides. If one mutation is introduced to a codon, it can occur at any of these three nucleotides. Every position already contains a specific nucleotide. The change can be made by replacing it with one of the three remaining bases. Therefore, a codon can be transformed into one of the nine possible codons by introducing a single base change. In this way, we obtain 9 ✕ 64 = 576 codon switches for all 64 codons. To generalize the applicability of codon switches, we need to also consider unchanged codons as codon switches, where both codons are identical. Therefore, we have 576 + 64 = 640 codon switches in the dictionary that can seamlessly represent any coding sequence. Each codon switch is assigned a unique numeric code from 0 to 639. To capture mutation identities adequately, we considered the sequence of surrounding codon switches. All protein-coding mRNA sequences (coding regions only) were extracted from the reference genome to construct these codon switch sequences. For each variant, we constructed a codon switch sequence based on the nucleotide triplets as observed in the corresponding reference sequence, except for the single codon switch difference due to the variant itself. For codon switches other than ones harboring variants, we considered identical nucleotide pairs as per the reference sequence. This is illustrated in Figure 1*A*. For the machine learning task, these codon switch sequences were converted into numeral sequences using their preassigned numeric codes. All analyzed variants were processed in this manner for embedding and other machine learning tasks.

### Continuous embedding of codon switches

A *skip-gram* (13) model with negative sampling was employed to learn continuous representations of codon switches. The *skip-gram* model learns embeddings by training a shallow neural network that attempts to predict a codon switch's context. The word whose context is being learned is referred to as a center codon switch. In general, the context (the surrounding nucleotides) of a codon switch is prohibitively long to predict; thus, we resort to the negative sampling approach. In the said approach, we define a small window (*ws*) around it for every codon switch in a sequence, and all codon switches in this region are termed context codon switches or positive samples. Further, some codon switches from outside the windows are randomly selected and termed the negative samples. The rate at which codon switches are sampled is called the negative sampling rate (*nsr*).

Theoretically, embeddings are learned by making every codon switch in a sequence a center codon switch. Of note, corner codon switches are also treated as center codon switches, but we look at only one side of the window to get the context. But, practically, in a large dataset with large sequences, the count of center codon switches is extremely high thus making it infeasible to use every instance of a codon switch as a center codon switch. Hence, we performed the subsampling to limit it. Since a codon switch sequence consists of codon switches that do not contain any nucleotide alteration except for one codon switch, the distribution of codon switches is heavily skewed toward the former type of codon switches. Thus, we first systematically squeeze the probability of frequent codon switches and inflate the probabilities of a nonfrequent switch. This increases the chances of nonfrequent codon switches getting selected as center codon switches. To systematically adjust the probability of codon switches, we use the following formula:

(1) $$probability\;of\;selecting\;a\;condon\;switch=\min\left(1,\left(1+\sqrt[{2}]{\frac{f}{\varepsilon }}\right).\frac{\varepsilon }{f}\right)$$

where *ε* = 0.001 and f are the codon switch frequency in the dataset kept aside for embedding. For each selected center codon switch, 2 ✕ ws tuples were constructed by pairing it with ws adjacent codon switches from both sides. Taken together, these tuples constituted the positive category. On the other hand, for every center codon switch, negative sampling was performed by pairing the center codon switch with random [((2 ✕ ws + 1) ✕ nsr)] or two codon switches, uniformly sampled from the codon switch dictionary. We used window size of 3 (ws) and negative sampling rate (nsr) of 0.2 for the construction of the dataset. For these values, a total of 219,418,024 tuples were generated. Out of this, 182,886,425 were generated as positive samples, and 36,531,599 were generated as negative samples.

In order to learn the 300-sized numeric vectors representing the 640 codon switches, we initialized a 640 ✕ *L*, where *L* = 300 sized matrix with random entries. To this end, we also simplify the training procedure of *skip gram*. We posed the problem of learning codon switch embedding as a classification. To build the dataset, we assigned a class label of 0/1 to all the codon switch pairs in the negative/positive sets (Fig. 1*B*). Then, a simple neural network was trained to classify between the tuples labeled as 0 or 1(Fig. 1*C*). Network S1 shows the network architecture to learn CRCSes. The input to the neural network was the concatenated vectors of length 2 ✕ *L*, corresponding to the pair of codon switches in each tuple. For a pair of embedding, we first compute the dot product of the two and then compute the sigmoid of the resulting value. The binary cross-entropy cost function was optimized on the output of the sigmoid unit. In total, the model has 192,002 trainable parameters. All parameters, except 2, are the learnable parameters from the embedding matrix. The other two parameters are for the last dense layer, where one of the parameters belong to the neuron's weight and the other one is for the bias of the layer. The ADAM (67) optimizer was used for optimization. The procedure was repeated for 200 epochs.

### Cross-chromosome sequence similarity analysis

To assess the diversity of chromosomes at the amino acid levels, we computed the proportion of unigram, bigram, and trigram of amino acids across all chromosome sequences. Here, a unigram is defined as a single amino acid. There were 21 unique unigrams; among those, 20 were amino acids and one representative unigram corresponding to a stop codon. Similarly, a bigram and trigram are defined as strictly ordered pairs and triplets of amino acids, respectively. In this manner, we obtained 441 unique bigrams and 9261 trigrams (including pairs and triplets of stop codon).

### Variant classification

The pretrained embeddings were used for classifying codon switch sequences. We labeled codon switch sequences stemming from ExAC and COSMIC as 0 and 1, respectively. To reduce the computational overhead, sequences of length less than 1500 were selected. Only genes with a minimum variant count of 200 (with alternate splicing) were retained, leading to 332 genes. These genes were then randomly split into 4-folds. These folds were created so that there are no common genes in train and validation splits.

A deep neural network was constructed to classify the sequences. The deep neural stack consisted of a nontrainable embedding layer, followed by two stacked bi-LSTM layers (68, 69), interleaved with one batch normalization layer (70). The bi-LSTM layers were followed by another batch normalization layer and a time-distributed dense layer. The time-distributed layer shared weights across all time-states in a sequence. However, the time-states did not communicate with each other. The time-distributed layer was followed by another batch normalization layer and an attention layer (71). The output layer is dense, and its neurons use a sigmoid activation function. In all, the model used 3,877,201 parameters, of which 194,600 were nontrainable or fixed. The embedding layer used pretrained codon switch embeddings, hence, marked as fixed, whereas other layers were initialized randomly, hence, marked as trainable. The Network was trained by minimizing a binary cross-entropy loss function. The ADAM (67) optimizer was used. A schematic of the network architecture is shown in Figure 3*A* and Network S2. The confidence scores for dbSNP and Met were generated after removing sequences that were part of the training set.

### Other methods for mutation annotation

We compare the performance of our architecture with two other methods, SIFT (20) and Polyphen2 (21), which annotates deleterious mutations. The SIFT algorithm's command-line version of the executable (for Linux) was downloaded from https://sift.bii.a-star.edu.sg/sift4g/AnnotateVariants.html. SIFT 4G database of chromosome X was downloaded from https://sift.bii.a-star.edu.sg/sift4g/public/Homo_sapiens/GRCh37.74/. We combined all four test folds into a single dataset to run the predictions. This combined dataset was sorted first on the chromosome, then on position, and then passed through the executable. The following command was used to annotate the mutation.

java -jar SIFT4G_Annotator.jar -c -i input_vcf.vcf -d sift_db -r output_folder

*SIFT_SCORE* column from the output file of SIFT was used for further analysis. The mutations with a low value of SIFT score represent the deleterious mutations. As per recommendation, if the predicted score is below 0.05, the mutation is deleterious. However, we considered these scores as continuous values and performed the analysis. To keep the scores in a similar range as our method, we subtracted SIFT scores from one before comparing.

We generated predictions from Polyphen2 using the web server available at http://genetics.bwh.harvard.edu/pph2/. We used the same dataset used for SIFT.

### Other available embeddings

To compare the efficacy of CRCS against other embeddings, we downloaded dna2vec (14) embeddings from https://github.com/pnpnpn/dna2vec. Dna2vec trains the word2vec model on *k*-mers of the human genome. We extracted 100 length embeddings of every codon (3-mer) of the human genome from the dna2vec model, thus resulting in an embedding matrix of 64 ✕ 100. This matrix is fed to the network in the embedding layer of our customized sequence classifier (Variant classification). Codon sequences in place of codon switch sequences were used for training with dna2vec embeddings.

### Other available architectures

We also compared the two widely used architectures developed for predicting functional effects of noncoding variants, namely DeepSea (9), DanQ (8), and one recently published architecture HeartENN (10). DeepSea and HeartENN are pure convolutional neural networks. In contrast, DanQ is a hybrid architecture having both convolutional and bidirectional LSTM layers. HeartENN has 90 neurons in the last layer, but we changed it to 919 neurons as in DanQ and DeepSea. Then, to make these architectures suitable to classify sequences into cancer and noncancer, we added one additional dense layer with single neuron and sigmoid activation at the end. Networks S3–S5 give the details about these architectures. Among these models, DanQ has the most parameters (206,177,959), followed by DeepSea (64,921,359). HeartENN has 58,525,559 parameters, out of which 760 are nontrainable. We used binary cross-entropy as the loss function to optimize these networks. We used RMSProp as the optimizer. One-hot–encoded protein-coding mRNA sequences are provided as input for training. Since convolutional neural networks work with fixed input size, we have padded all the variable length sequences to 4500 (1500 length codon switch sequence) with 0s.

### Comparing cBioPortal predictions with dbSNP predictions

In order to extract the significant genes for different cancer types, we compared the prediction scores generated on the cBioPortal with the prediction scores generated on the dbSNP. Fig. S10 shows the prediction scores on cBioPortal data for various cancer types. For the cBioPortal data, we grouped predictions on cancer type and genes. For the dbSNP database, the predictions were grouped based on genes alone. The cutoff for the group size was set to 5. We compared the groups obtained using cBioPortal and dbSNP. The Mann-Whitney U-test with alternate hypothesis cBioPortal > dbSNP was used to determine the statistical significance of genes. For a given cancer type, *p*-values of all genes were collected and corrected using the *holm-sidak* method (Supplementary File 3). The resulting gene sets were used for Gene Ontology analysis (33).

To perform the driver gene analysis using the selected gene sets, we first selected the genes that were present in most cancer types. Genes occurring in ≥10 cancer types were selected for analysis. This resulted in 32 significant genes. Among the selected cancer types, we removed those cancer types that had ≤5 genes, resulting in 25 cancer types.

### Parameter choices

#### Embedding parameters

window size (*ws*) and *nsr* were selected as 3 and 0.2, respectively.

#### Model parameters

We selected the regularization hyperparameters, attention regularization, and other layer weight regularization using 4-fold cross validation. We used grid search to search over the hyperparameter space. Neural Network architecture was selected using a systematic trial and error approach.

#### Other annotation algorithms

We used SIFT and Polyphen2 with standard parameters.

#### dna2vec and other deep learning algorithms

We have used the same architecture proposed by the authors of those architectures.

### Patient-level cumulative BLAC scores for survival analysis

We created a cumulative BLAC score for every patient by combining the scores for the mutations found on chromosome X in the patient. This cumulative score is designed to give exponentially higher weightage to high BLAC scores. The cumulative BLAC score will be termed as BLACs score hereafter. The BLACs score for a patient *P* is given by

(2) $$BLACs\;score_{P}=\frac{\sum_{i=1}^{i=N}2^{k.\left(BLAC\;scor{e_{i}}^{p}-1\right)}}{N}$$

where *N* is the total number of mutations on chromosome X on patient *P* and *k* is a scaling factor. We obtained the best performance when *k* is set to 4.

The cBioPortal (62) dataset was used for survival analysis. As discussed in the earlier sections (Classifying cancerous and noncancerous mutations and Pruning of the coding variants), the filtering steps applied to select the candidate mutations are (i) synonymous mutations and indels were removed. (ii) All noncoding mutations were removed. (iii) All the mutations that were part of ExAC or COSMIC databases were also dropped since these mutations were present in the training data. After these steps, we were left with 147,048 unique mutations, which collectively span across 293 ONCOTREE cancer subtype codes (72) and 14,349 patients. Further, we selected only those patients who had ≥5 mutations left after filtering. Then, these patients were grouped as per their cancer types. Any cancer type having less than 100 patients was also dropped from the analysis. After all the filtering steps, we were left with eight cancer types. We computed the BLACs scores for every patient in these cancer types. For every cancer type, the patients were divided into two groups for every cancer type by thresholding BLACs scores. The optimal threshold for every cancer type was identified using χ^2^ statistics (73). The *survfit* and *surfdiff* functions from survival package in R (v4.1.3) were used to perform the analysis.

Of note, after all the filtering, there was not enough data left to perform disease-free survival. Thus, we only performed survival analysis on overall survival data.

## Data availability

No new data was generated as part of the study. All the experiments for the article were performed in python 3.7.2, unless specified. All the deep neural networks were implemented using TensorFlow 2. Software and code developed for this study are present at https://github.com/Aashi-Jindal/CRCV.

## Supporting information

This article contains supporting information (8, 9, 10, 14).

## Conflicts of interest

The authors declare that they have no competing interests.

## Footnotes

† Mathematically, total codon switch count is given as n^c^(^c^C_1_✕ ^n − 1^C_1_ + ^c^C_c_) where codon length, *c* = 3, and number of nucleotides, *n* = 4.
