# Supplementary material for: A new deep learning technique reveals the exclusive functional contributions of individual cancer mutations
Source: J Biol Chem. 2022 Jun 24;298(8):102177. doi: 10.1016/j.jbc.2022.102177 (PMC9304782; doi:10.1016/j.jbc.2022.102177)
Supplement: Supplementary File 1 [file mmc1.pdf]

Supplementary Material

A new deep learning technique reveals the exclusive functional contributions of individual cancer mutations

Prashant Gupta[1,+], **Aashi Jindal**[1,+], **Gaurav Ahuja**[2], **Jayadeva**[1,*], **and Debarka Sengupta**[2,3,4,*]

[1]Department of Electrical Engineering, Indian Institute of Technology Delhi, Hauz Khas, Delhi 110016, India

[2]Center for Computational Biology, Indraprastha Institute of Information Technology, Delhi 110020, India

[3]Department of Computer Science and Engineering, Indraprastha Institute of Information Technology, Delhi 110020, India

[4]Center for Artificial Intelligence, Indraprastha Institute of Information Technology, Delhi 110020, India

[*]To whom correspondence should be addressed. Tel: +91 11 26907446; Email: debarka@iiitd.ac.in , Correspondence may also be addressed to Jayadeva. Tel: +91 11 26591087; Email: jayadeva@ee.iitd.ac.in

[+]These authors contributed equally to this work.

S1 Comparison of CRCS based approach with the existing best practice architectures

As an alternative approach to embedding, we used `dna2vec`[1], which learns numeric embeddings for variable-length *k*-mers from the reference human genome sequence. Notably, it does not provide the strategy to learn from SNVs curated by databases such as ExAC. We encoded the codons from the altered sub-sequence using the `dna2vec` representation. These encoded sequences are then fed to our custom architecture, BLAC. To keep the comparison fair, we used the same fold as used for training with BLAC. Fig. S7A, B shows the comparison of the performances of the model trained using these two embeddings. It is evident from the figure that dna2vec representation could not discriminate between the cancerous and non-cancerous sequences (Mann-Whitney U-test P-value $= 1$).

To further validate the performance of our model, three different architectures, namely, `DanQ`[2], `DeepSea`[3], and `HeartENN`[4], were compared. One additional single neuron layer with sigmoid activation (logistic layer) was added at the end to enable these architectures to classify the sequences into cancerous and non-cancerous categories. As prescribed by the authors of these models, one-hot encoded ACTG sequences were provided as input. On the other hand, our model was trained with the CRCS embeddings. Fig. S7C, D shows the comparison of the performances of these models. Our model performed best (AP=0.78) followed by `DeepSea` (AP=0.77). `DanQ` could not differentiate between cancerous and non-cancerous sequences. (Mann-Whitney U-test P-value $= 1$). Although `HeartENN` has differing distribution (Mann-Whitney U-test P-value $= 9.08 \times 10^{-9}$), it was not able to properly differentiate between the two classes (AP=0.72)[1]

[1] F1-score, specificity, and sensitivity were not reported for these methods since the prediction range for these methods are very small (Fig. S7B, D). It was not evident which threshold value should be chosen to justify the result. We instead report average precision, which is amortized over different threshold values.

S2 Extended Switch Dictionary

In Section 4.3 we discussed the steps to construct the codon switch dictionary containing only single point mutation. We obtained 640 unique switches corresponding to single base substitutions. This number also includes the cases with no mutations. Following similar steps, double and triple base substitutions can be modeled by 1728 switches each.

To include the insertion and deletions into the dictionary, we need to introduce another character, \$, to represent a space. Now, to construct switches that represent insertions, the reference codon will have \$ at the point of insertions. For example, in a nucleotide sequence AA, if there is an insertion of T at the beginning, the constructed switch will have the form $\$AA \rightarrow TAA$. Similarly, other switches can be constructed for insertions. Assume that a sequence $TACGTCT$ is inserted between AA, then we assume that the reference sequence is $A\$\$\$\$\$\$\$A$ and it will be converted into the codon switch sequence with $A\$\$ \rightarrow ATA$, $\$\$\$ \rightarrow CGT$, and $\$\$A \rightarrow CTA$ codon switches. All the switches representing deletions can also be constructed by following the same steps, except that the space character is introduced in the altered codon. Switches representing complex insertions and deletions can also be constructed where space character appears in both reference and altered codons. Taken together, the cumulative number of codon switches rises to $15,625$ after considering complex mutations and indels.

To do a fair analysis, we computed the frequency of different codon switches on the inflated mutation counts from the ExAC DB (section 4.2). We obtained an average of 495 variants associated with each of the 640 codon switches representing single base substitution. Notably, all the codon switches with one alteration were present in the datasets. From the same database, we found at least one variant for 66 out of the 1728 possible coding switches representing double mutations (consecutive and non-consecutive). The average per switch variants obtained from the database was $\sim$1.5. For the triple substitutions, an average of 1.7 variants were spotted for 53 (out of 1728) switches. Statistics for the remaining variant types can be found in Table S2.

These double/triple substitutions and insertion and deletions frequencies are not enough to learn the embeddings. Given the sufficient amount of data, the dictionary constructed after all inclusions can be used to learn embeddings. Counts for all the switches are shared in Supplementary File 3.

Method	Specificity	Sensitivity	F1-score
BLAC	0.518	0.620	0.686
SIFT	0.556	0.561	0.633
Polyphen2	0.632	0.499	0.585

Table. S1. Specificity, Sensitivity and, F1-score values at the threshold of 0.9. This value of threshold was chosen since predictions of all the algorithms in Fig. S4 are skewed toward high values. These metrics are computed on the predicted scores on mutations reported in ExAC and COSMIC databases.

Mutation type	Total switches	Available switches in data	Average frequency of available switches
Single base substitutions	640	640	495
Double base substitutions (consecutive & non-consecutive)	1728	66	1.5
Triple base substitutions	1728	53	1.7
Insertions	3904	290	10
Deletions	3904	357	25
Complex Indels	3721	0	0

Table. S2. Frequencies of switches in different variant type formed using ExAC dataset.

Network S1 Network architecture to learn the Continuous Representation of Codon Switches (CRCS)

index1 ← embedding array index of first switch in the pair
index2 ← embedding array index of second switch in the pair
label ← label of the switch pair
emb1 = EmbeddingMatrix(index1)
emb2 = EmbeddingMatrix(index2)
dot = **Dot**([*emb1*, *emb2*])
output = **Dense**(*dot*, neurons=1, activation='sigmoid')
cost = **BinaryCrossEntropy**(*output*, label)

Network S2 Customized neural network for sequence classification - Bi-LSTM with Attention & CRCS (BLAC)

seq ← Switch sequence
label ← label of the sequence
embeddings = **Embeddings**(seq)
bi1 = **BiLSTM**(*embeddings*, neurons=300, how='cascade')
ba1 = **BatchNorm**(*bi1*)
bi2 = **BiLSTM**(*ba1*, neurons=300, how='cascade')
ba2 = **BatchNorm**(*bi2*)
td = **TimeDistributedDense**(*ba2*, neurons=100)
ba3 = **BatchNorm**(*td*)
at = **Attention**(*ba3*)
output = **Dense**(*at*, neurons=1, activation='sigmoid')
cost = **BinaryCrossEntropy**(*output*, label)

Network S3 Modified DeepSea Neural Network

```
seq ← One-hot encoded protein coding mRNA Sequence
label ← label of the sequence
conv1 = Conv1D(seq, filter=320, kernelsize=8, padding='valid', activation='relu')
mp1 = MaxPool1D(conv1, poolsize=4, strides=4)
d1 = Dropout(mp1, 0.2)
conv2 = Conv1D(d1, filter=480, kernelsize=8, padding='valid', activation='relu')
mp2 = MaxPool1D(conv2, poolsize=4, strides=4)
d2 = Dropout(mp2, 0.2)
conv3 = Conv1D(d2, filter=960, kernelsize=8, padding='valid', activation='relu')
mp3 = MaxPool1D(conv3, poolsize=4, strides=4)
d3 = Dropout(mp3, 0.5)
f = Flatten(d3)
D1 = Dense(f, neuron=919, activation='relu')
output = Dense(D1, neurons=1, activation='sigmoid')
cost = BinaryCrossEntropy(output, label)
```

Network S4 Modified DanQ Neural Network

seq ← One-hot encoded protein coding mRNA Sequence
label ← label of the sequence
conv1 = **Conv1D**(seq, filter=320, kernelsize=26, padding='valid', activation='relu')
mp1 = **MaxPool1D**(*conv1*, poolsize=12, strides=13)
d1 = **Dropout**(*mp1*, 0.2)
b1 = **BiLSTM**(*d1*, neurons=320)
d2 = **Dropout**(*b1*)
f = **Flatten**(*d2*)
D1 = **Dense**(*f*, neurons=925, activation='relu')
D2 = **Dense**(*D1*, neurons=919, activation='relu')
output = **Dense**(*D2*, 1, activation='sigmoid')
cost = **BinaryCrossEntropy**(*output*, label)

Network S5 Modified HeartENN Neural Network

```
seq ← One-hot encoded protein coding mRNA Sequence
label ← label of the sequence
conv1 = Conv1D(seq, filter=60, kernelsize=8, padding='valid', activation='relu')
conv2 = Conv1D(conv1, filter=60, kernelsize=8, padding='valid', activation='relu')
mp1 = MaxPool1D(conv2, poolsize=4, strides=4)
b1 = BatchNorm(mp1)
conv3 = Conv1d(b1, filter=80, kernelsize=8, padding='valid', activation='relu')
conv4 = Conv1d(conv3, filter=80, kernelsize=8, padding='valid', activation='relu')
mp2 = MaxPool1D(conv4, poolsize=4, strides=4)
b2 = BatchNorm(mp2)
d1 = Dropout(b2, 0.4)
conv5 = Conv1d(d1, filter=240, kernelsize=8, padding='valid', activation='relu')
conv6 = Conv1d(conv5, filter=240, kernelsize=8, padding='valid', activation='relu')
b3 = BatchNorm(conv6)
d2 = Dropout(b3, 0.6)
f = Flatten(d2)
D1 = Dense(f, neurons=919, activation='relu')
output = Dense(D1, neurons=1, activation='sigmoid')
cost = BinaryCrossEntropy(output, label)
```

References

1. Ng, P. dna2vec: Consistent vector representations of variable-length k-mers. *arXiv preprint arXiv:1701.06279* (2017).

2. Quang, D. & Xie, X. Danq: a hybrid convolutional and recurrent deep neural network for quantifying the function of dna sequences. *Nucleic acids research* **44**, e107–e107 (2016).

3. Zhou, J. & Troyanskaya, O. G. Predicting effects of noncoding variants with deep learning–based sequence model. *Nat. methods* **12**, 931–934 (2015).

4. Richter, F. *et al.* Genomic analyses implicate noncoding de novo variants in congenital heart disease. *Nat. genetics* **52**, 769–777 (2020).

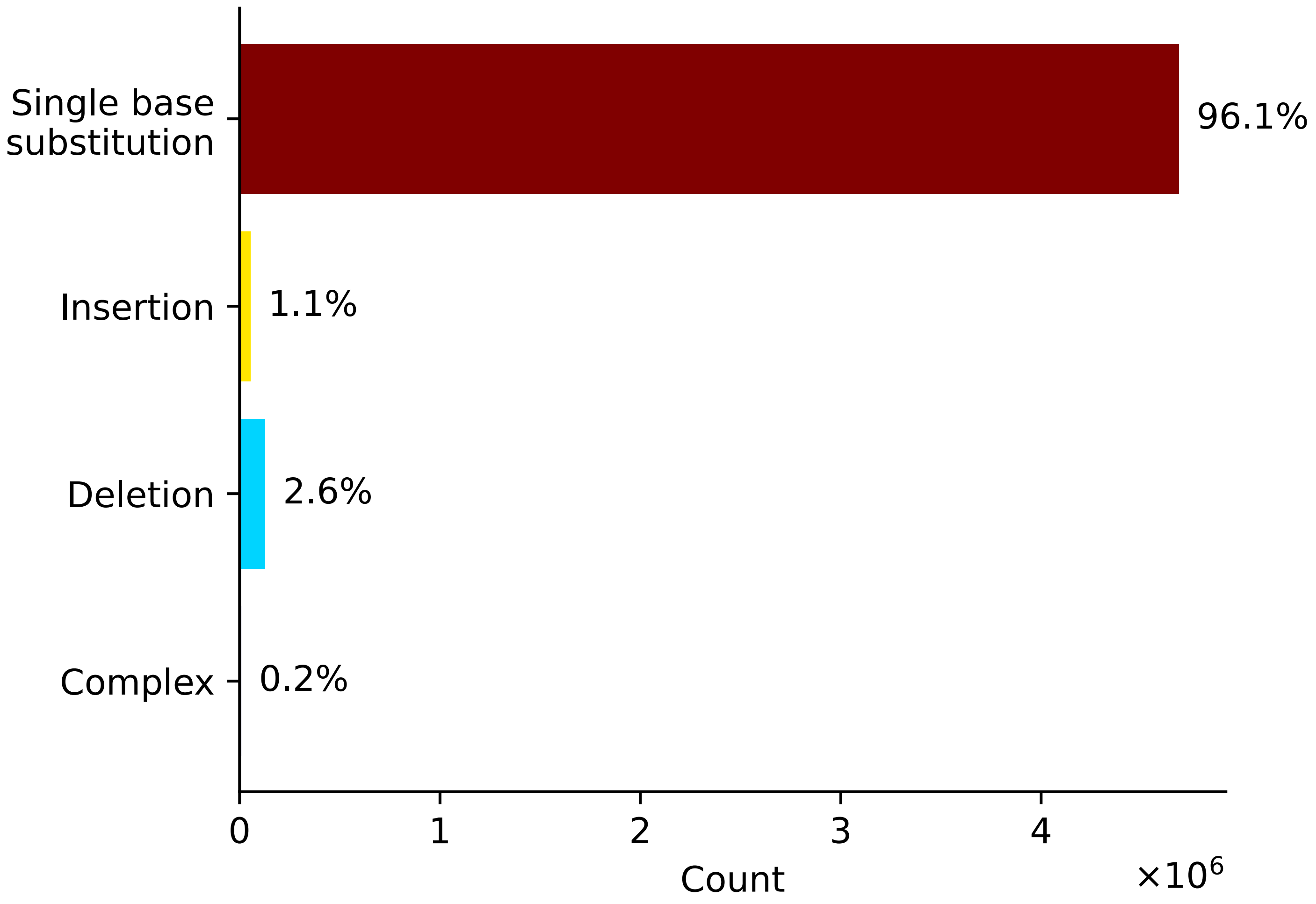

Fig. S1. Variant distribution in COSMIC data (v89).

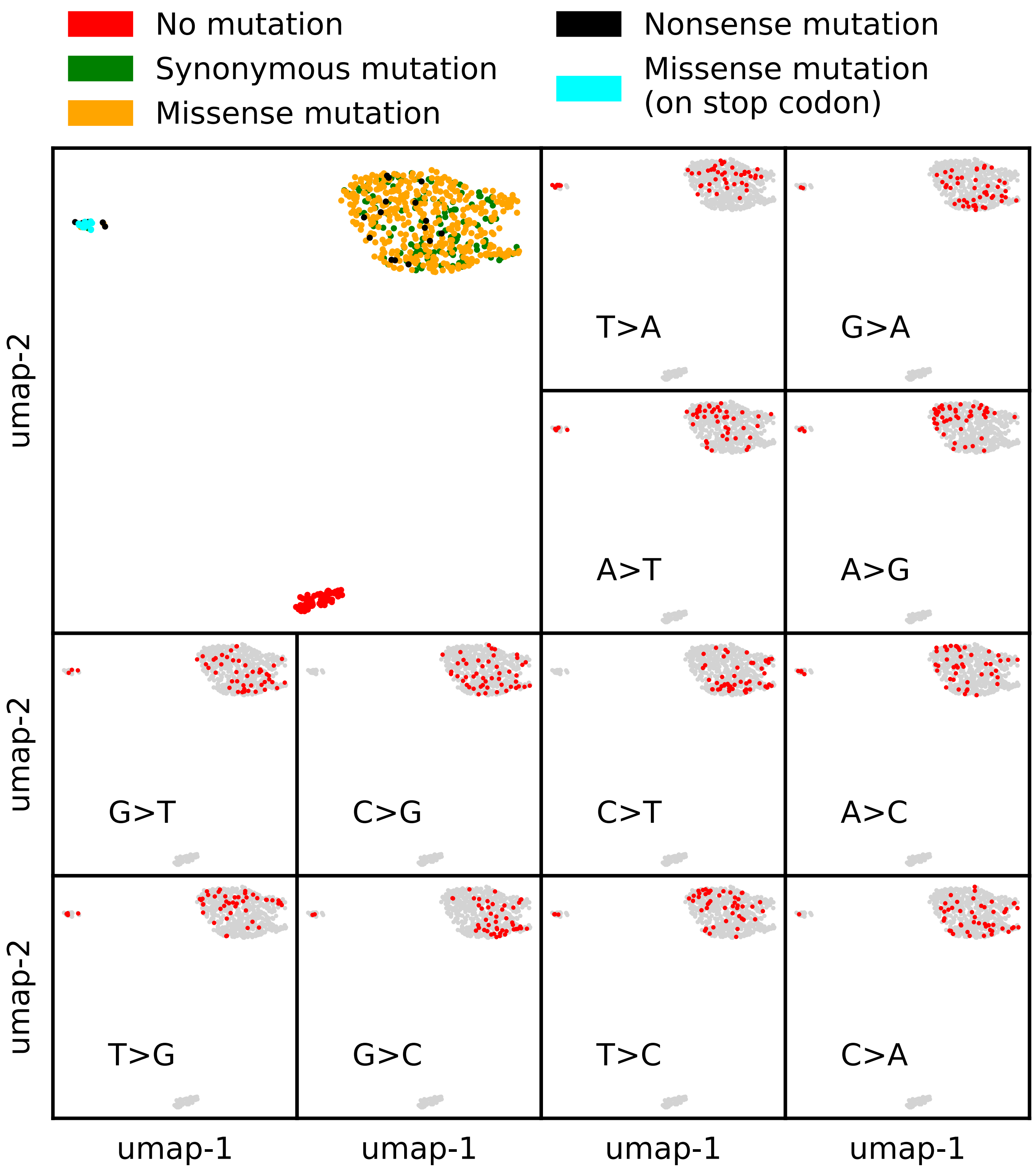

Fig. S2. UMAP projections of the switch embeddings. The top left plot shows the annotation of the switches based on the type of mutation. Switches without any mutations form a separate cluster. Mutations on the stop codons are also clustered separately. Synonymous and Nonsense mutations fall in the overlapping clusters. The remaining plots show the positions of the switch with a mutation in the clusters. Switches with complementary mutations tend to fall farther away in the cluster.

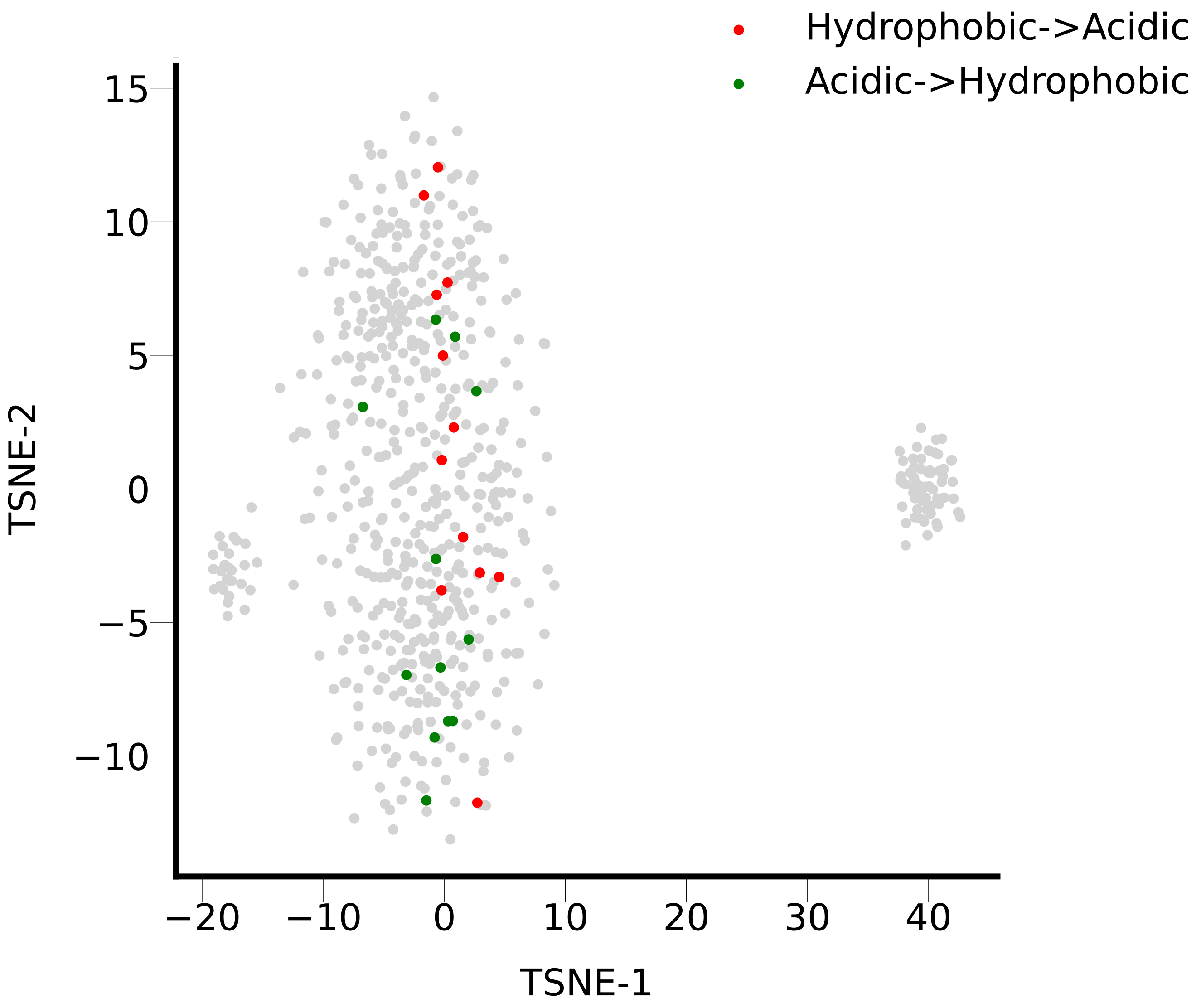

Fig. S3. Arrangement of switches, where the characteristic of amino acids change from acidic to hydrophobic and hydrophobic to acidic. Both the changes seem to get localized in different clusters.

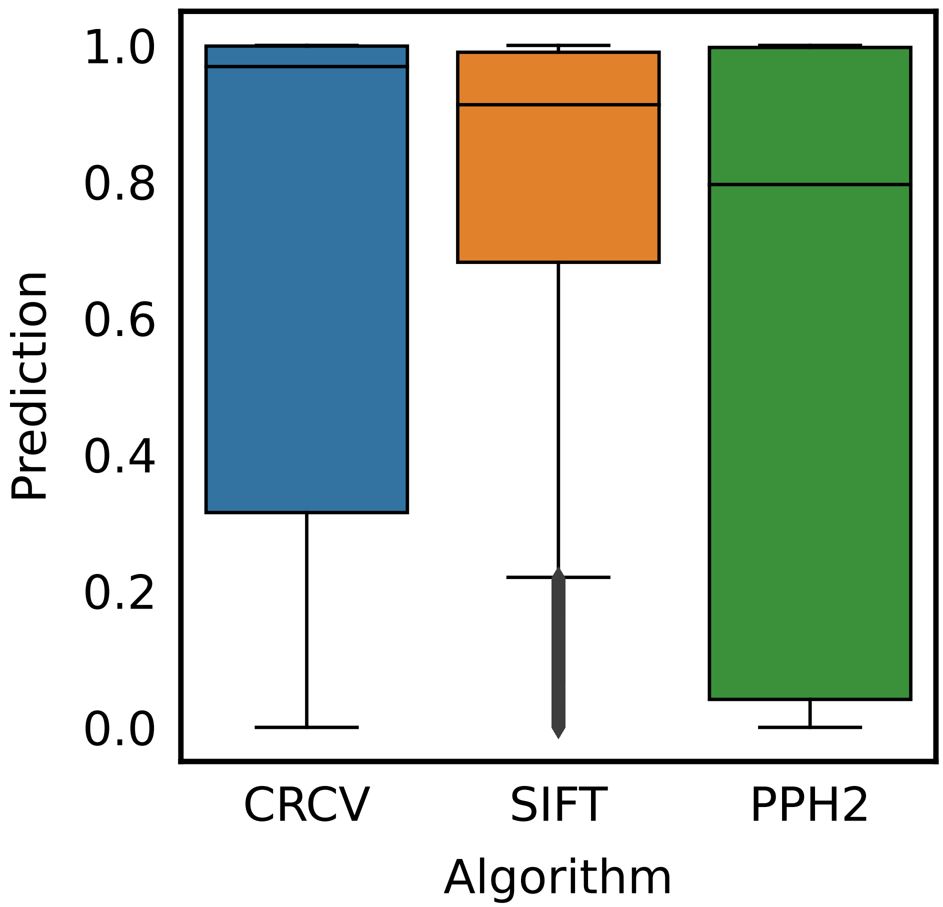

Fig. S4. Distribution of overall predicted scores by different algorithms.

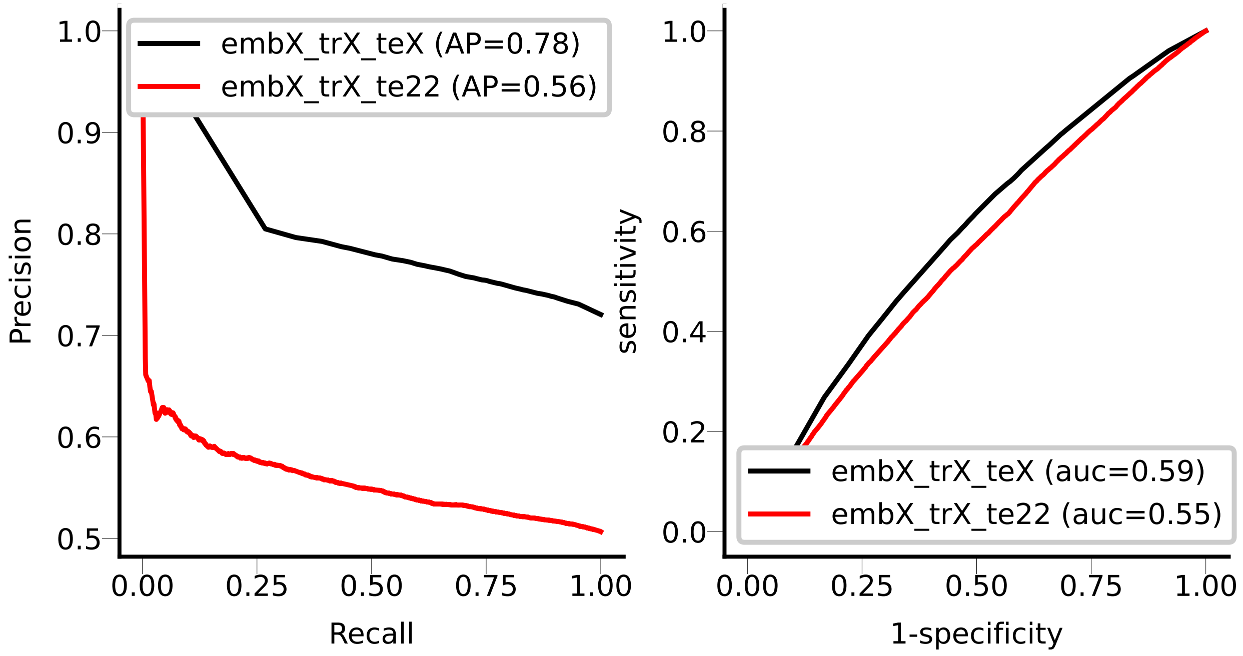

Fig. S5. Evaluation of model trained on chromosome X against chromosome 22. As expected, model performance deteriorated.

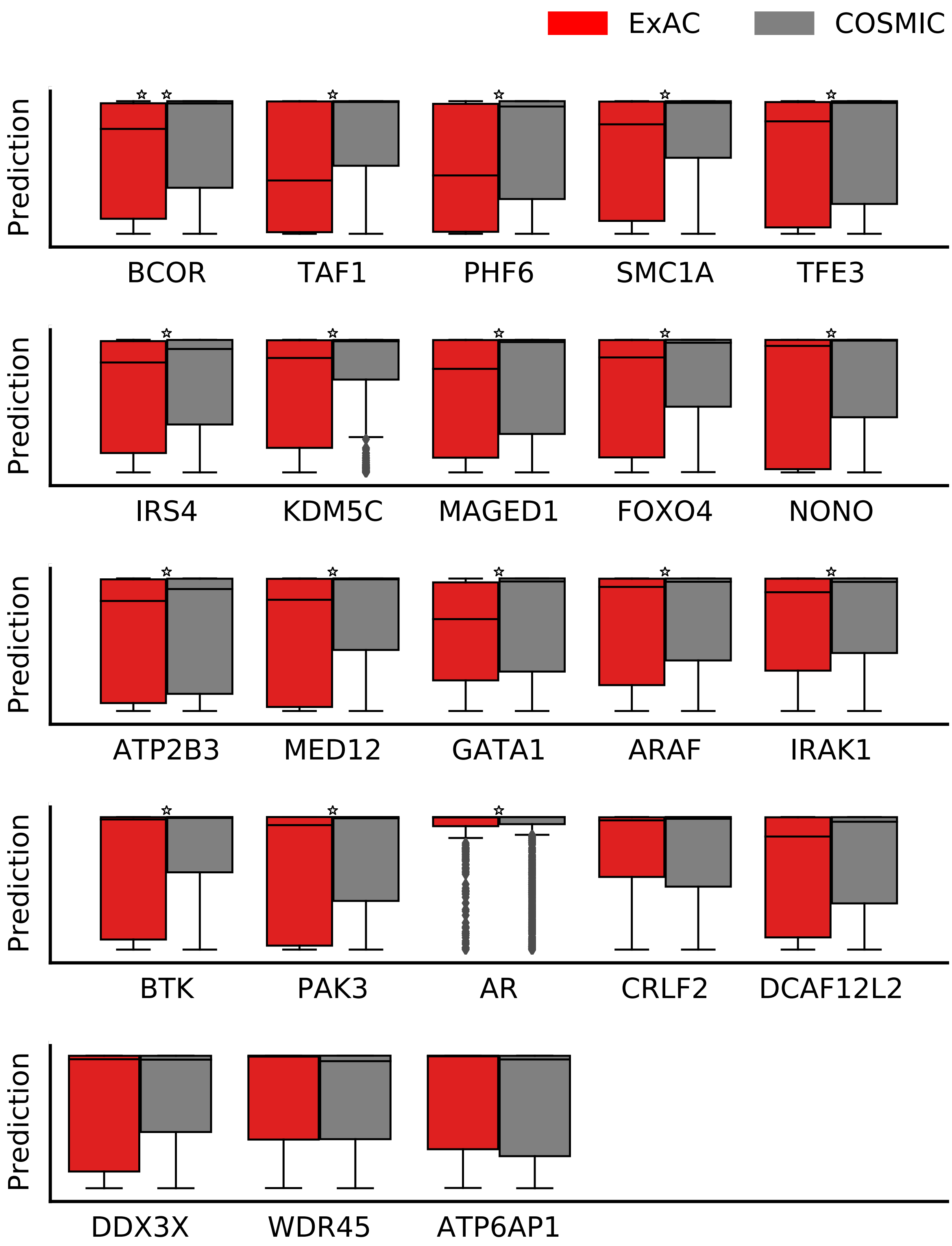

Fig. S6. Boxplots show the distribution of the prediction scores assigned to ExAC and COSMIC alterations for the known driver genes from the validation set (across all folds), except top 10. Top 10 values are present in Fig. 3. In the figure, 5 stars represent a *P*-value less than $5e^{-15}$. Values in the range $[5e^{-15}, 5e^{-12})$ are represented by 4 stars. Similarly, values in the range of $[5e^{-12}, 5e^{-9})$, $[5e^{-9}, 5e^{-6})$, and $[5e^{-6}, 5e^{-2})$ are represented by 3, 2, and 1 stars, respectively

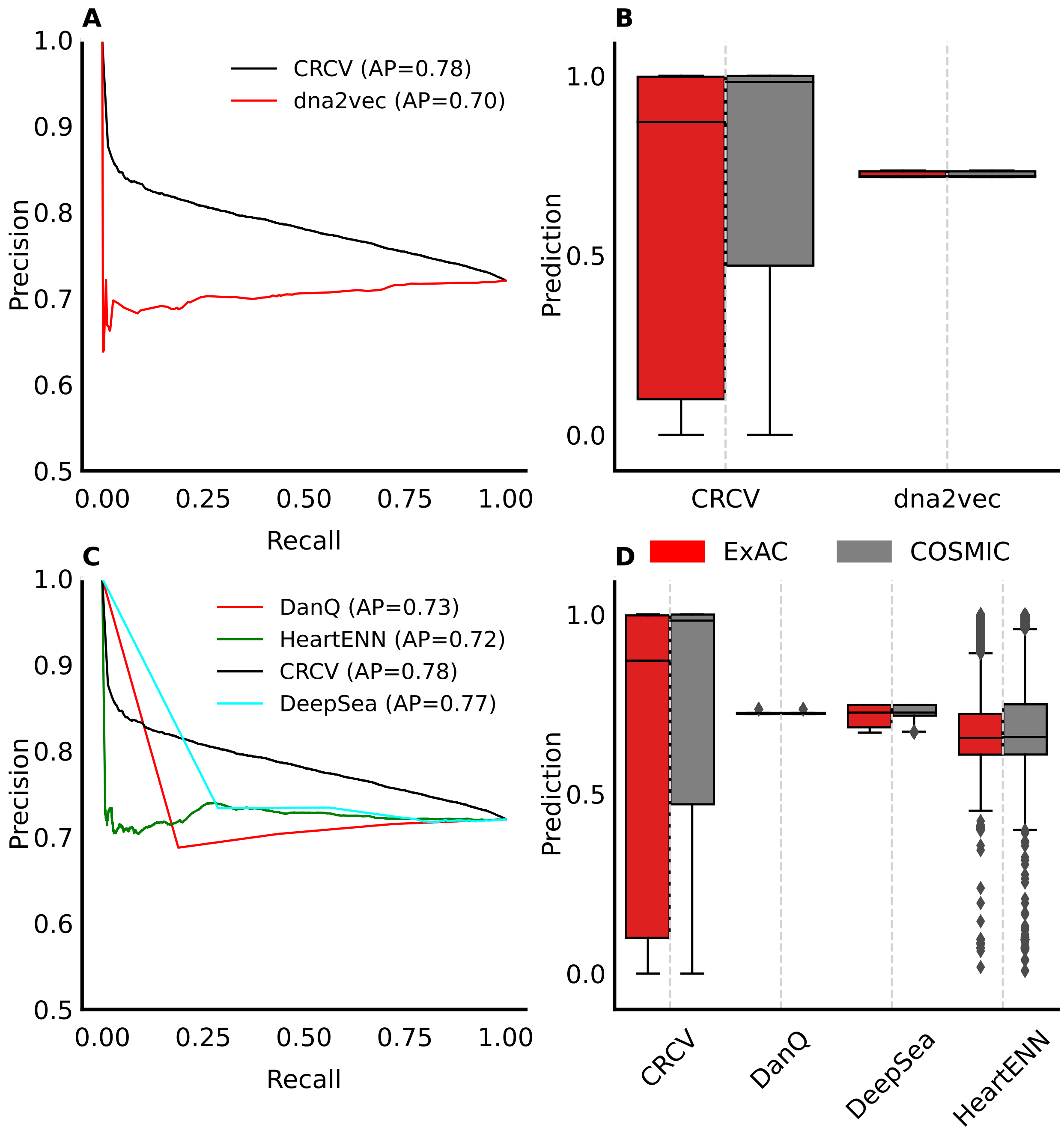

Fig. S7. Performance comparison of BLAC scores. A) Precision-Recall plot of the predictions obtained from the model trained with CRCS embeddings and dna2vec embeddings. B) Comparison of the distribution of scores obtained from the model. dna2vec does not have any discriminating power (Mann-Whitney U-test P-value = 1). H) Precision-Recall plot of the predictions obtained from other deep learning models, DanQ, DeepSea, and HeartENN. Compared to our proposed model trained with CRCS, other models have inferior performance. C) Comparison of the distribution of scores obtained by models. DanQ does not have any discriminating power (Mann-Whitney U-test P-value $= 1$). Other models have a different distribution of scores on ExAC and COSMIC. Mann-Whitney U-test P-value for DeepSea and HeartENN is 2.7×10^{-20} and 9.08×10^{-9} respectively. Our model with CRCS has the most differentiating power (Mann-Whitney P-value is almost near 0).

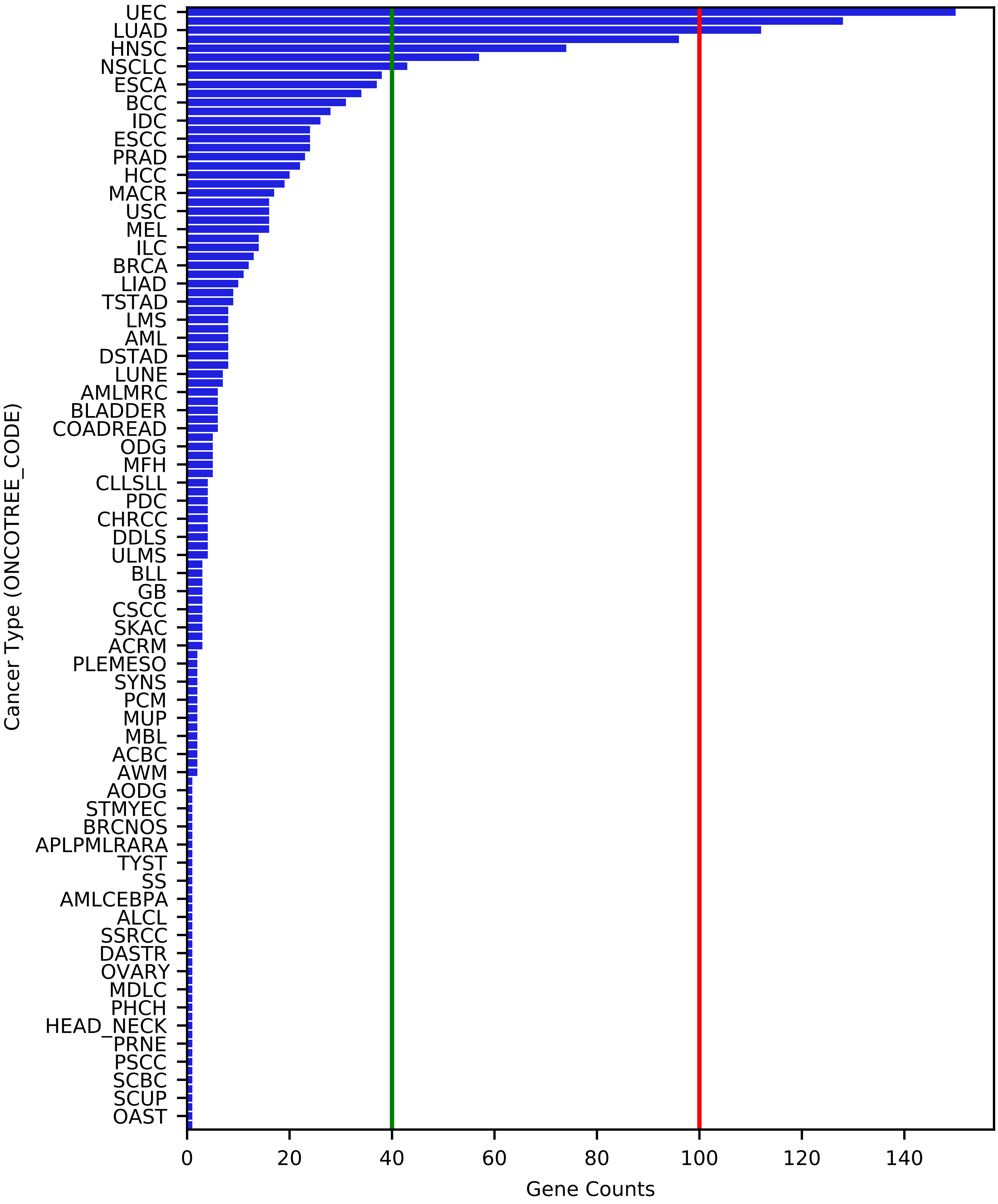

Fig. S8. The size of the gene group for different cancer types shown in the blue bars. The output of the Go-Term analysis of the cancer types above the red line is shown in the Fig. 6. Out of the Go-Term analysis of the cancer types above the green line is shown in the Fig. S9.

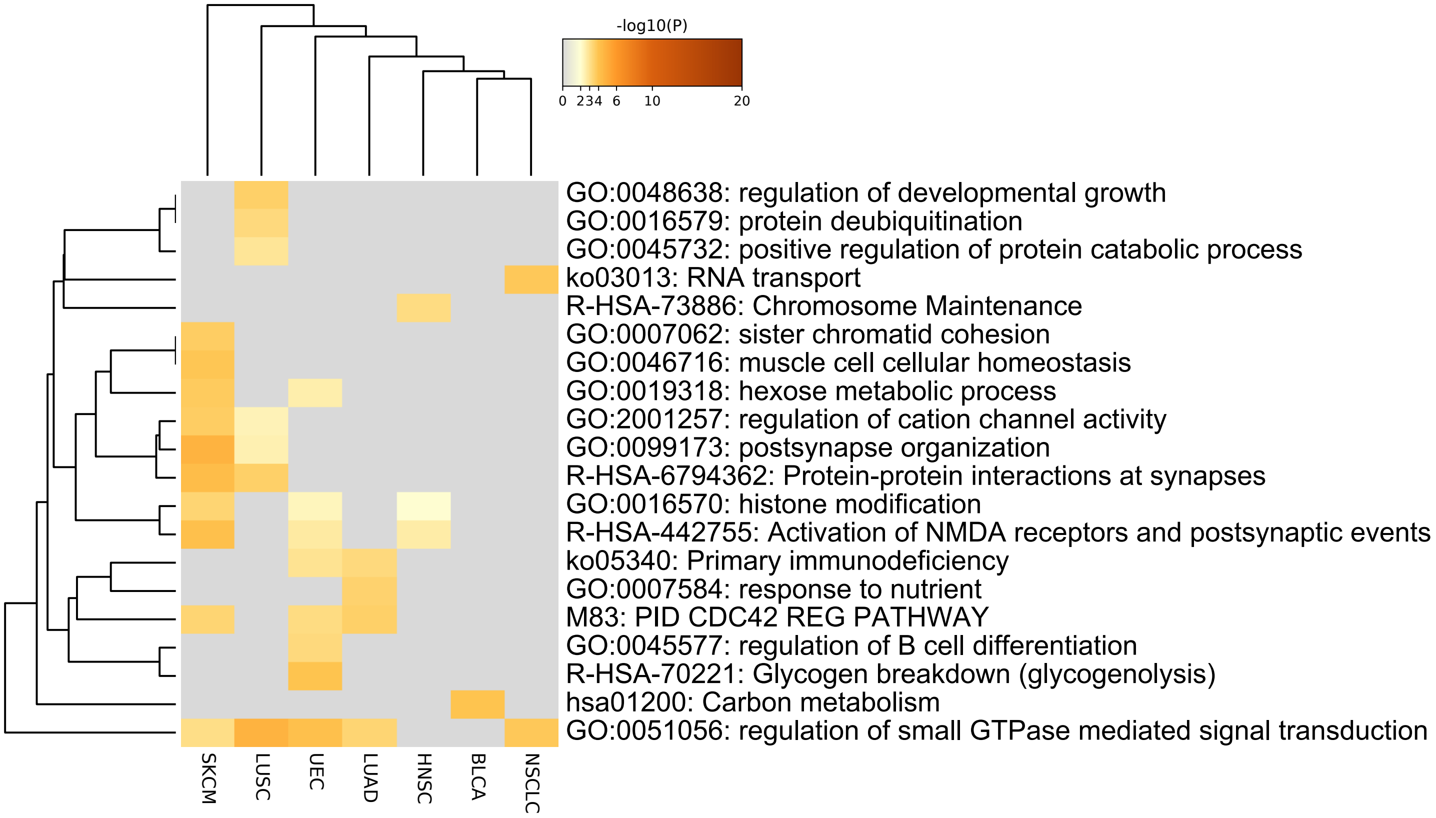

Fig. S9. Heatmap depicting the cluster-wise enrichment of the prominent biological functions in the indicated cancer types. Of note, the selected cancer types harbored the number of mutational genes identified using BLAC. Cancer types include Skin Cutaneous Melanoma (SKCM), Lung Adenocarcinoma (LUAD), Undifferentiated Endometrial Carcinoma (UEC), Lung Squamous Cell Carcinoma (LUSC), Head-Neck Squamous Cell Carcinoma (HNSC), Urothelial Bladder Carcinoma (BLCA), and Non-small-cell Lung Carcinoma (NSCLC). The scale bar represents the negatively log-transformed (base 10) *P*-values.

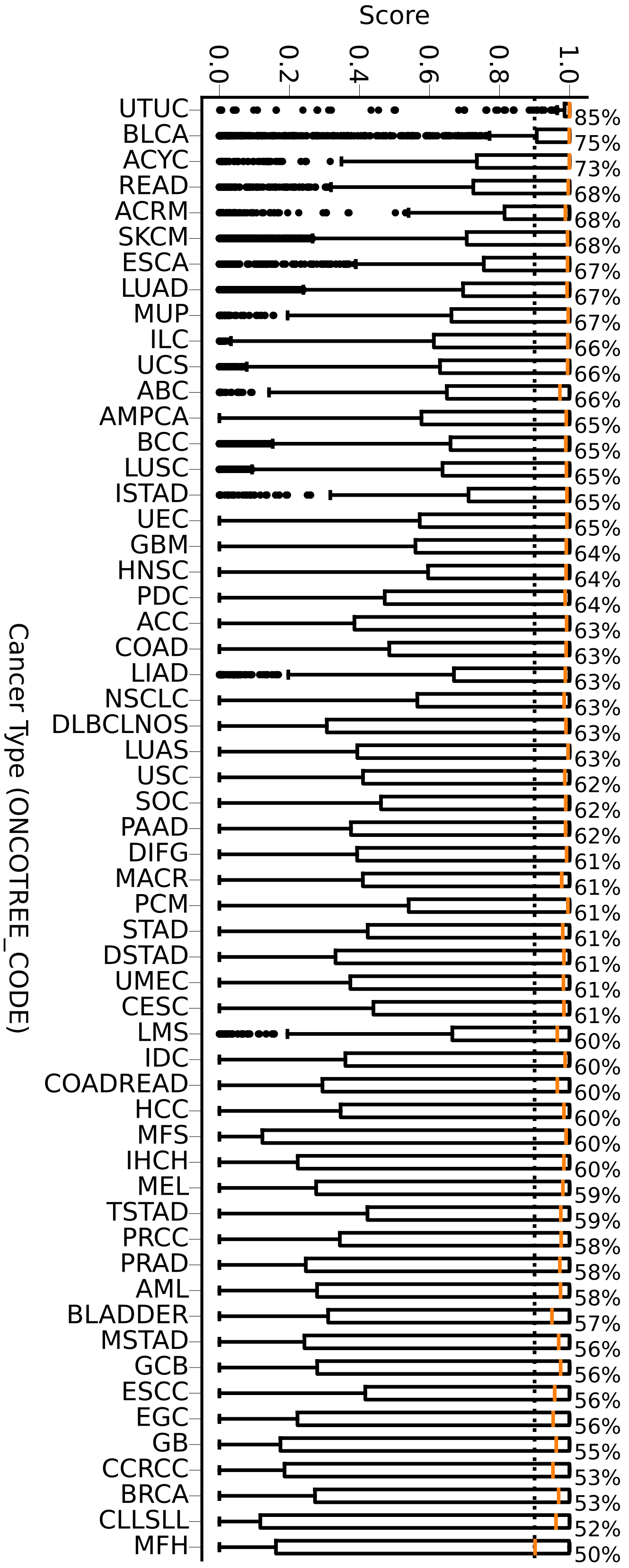

Fig. S10. Distribution of prediction scores on cBioPortal data. For almost all the cancer types median of the prediction score lies above 0.9. The percentage of prediction above the 0.8 threshold is shown with every boxplot.
